# Self-filtering based on the fault ride-through technique using a robust model predictive control for wind turbine rotor current

**DOI:** 10.1038/s41598-023-51110-3

**Published:** 2024-01-22

**Authors:** Abdelkader Achar, Youcef Djeriri, Habib Benbouhenni, Ilhami Colak, Mihai Oproescu, Nicu Bizon

**Affiliations:** 1 Intelligent Control and Electrical Power Systems Laboratory, Department of Electrotechnics, Faculty of Electrical Engineering, Djillali Liabes University, Sidi Bel-Abbes, Algeria; 2 Faculty of Engineering and Architecture, Department of Electrical and Electronics Engineering, Nisantasi University, 34481742 Istanbul, Turkey; 3 The National University of Science and Technology POLITEHNICA Bucharest, Pitești University Centre, 110040 Pitesti, Romania; 4 Doctoral School, Polytechnic University of Bucharest, 313 Splaiul Independentei, 060042 Bucharest, Romania; 5 ICSI Energy, National Research and Development Institute for Cryogenic and Isotopic Technologies, 240050 RamnicuValcea, Romania

**Keywords:** Energy science and technology, Engineering

## Abstract

This paper studies the possibility of connecting Wind Farms (WF) to the electric grid with the use of finite space model predictive command (FS-MPC) to manage wind farms to improve the quality of the current output from the doubly-fed induction generator (DFIG) with considering fault ride-through technique. This proposed system can generate active power and enhance the power factor. Furthermore, the reduction of harmonics resulting from the connection of non-linear loads to the electrical grid is achieved through the self-active filtering mechanism in DFIGs-WF, facilitated by the now algorithm proposed. FS-MPC technique has the ability to improve system characteristics and greatly reduce active power ripples. Therefore, MATLAB software is used to implement and verify the safety, performance, and effectiveness of this designed technique compared to the conventional strategy. The results obtained demonstrated the effectiveness of the proposed algorithm in handling the four operational modes (Maximum power point tracking, Delta, Fault, and Filtering). Additionally, the suggested technique exhibited flexibility, robustness, high accuracy, and fast dynamic response when compared to conventional strategies and some recently published scientific works. On the other hand, the THD value of the current was significantly reduced, obtaining at one test time the values 56.87% and 0.32% before and after filtering, respectively 27.50% and 0.26% at another time of testing, resulting in an estimated THD reduction percentage of 99.43% and 99.05%, respectively. These high percentages prove that the quality of the stream is excellent after applying the proposed strategy.

## Introduction

The continuous progress in the research field, especially in the field of electronics and the large consumption of electrical energy, led to the search for electronic elements such as semiconductors with high flexibility and high voltages to use them in high energies such as 1.5 MW or 20 MW. In the field of power generation, power transformers are widely relied upon, as these transformers consist of very sensitive electronic components^1^. The poor performance of an inverter used in power systems is among the things that lead to several problems such as increased power ripples, poor power quality and the existence of harmonics, which is undesirable, causing disturbances in the network and thus an operation malfunction of other devices and systems. Also, among other problems, especially in the field of high energies, there are no electronic elements that can withstand high voltages and large current intensities, which increases frequent malfunctions. The latter increases the maintenance bill, which is undesirable. Therefore, choosing these electronic elements that make up the inverter and the power systems is one of the priorities that must be taken care of in order to obtain a high-performance inverter and a highly efficient power system. In addition, the use of power transformers in the field of renewable energies is accompanied by a set of challenges and difficulties due to their classification as non-linear loads, which leads to undesirable effects on the electrical network. The latter must be characterized by several features such as stability and durability for the good operation of electrical systems^2^.

Non-linear loads absorb non-sinusoidal currents, acting as generators of reactive power (*Qs*) harmonics causing power factor (PF) degradation, which is undesirable. As it is known, the PF should be as close as possible to the ideal value of 1, as this value implies a good generated power quality. Consequently, if the value is less than 1, then the system does not work optimally and results in a low quality of the generated energy, which creates problems and faults in the network^3^. So, through this factor one can know the level of efficiency of the energy system and improvement of energy characteristics, as well as the degree of overcoming the problem of ripples. Therefore, it is necessary to pay attention to this factor and improve it. To overcome this problem and ensure a high-quality of the power transmitted to the network, parallel active filters are used as a suitable solution for power generation systems^4^. These filters are devices whose role is to improve energy quality and overcome problems in energy systems, as they consist of electronic elements such as relays. Also, these filters need control strategies such as reflectors. As is known, the inverter can be used as a power filter and therefore the concept of a single-phase filter and a three-phase filter appear in the specialized literature. These filters play a major role in improving power quality, cleaning the network, reducing harmonic currents, and effectively compensating *Qs*, thus obtaining a highly stable electrical network^5^. The stability of systems or networks is one of the most prominent problems in energy systems, as this characteristic must be taken care of and achieved in order to obtain good operation and excellent results in terms of power quality and current.

Power generation systems are different and dependent on energy sources used in generation, where traditional sources such as gas and coal are found and these are undesirable and cause environmental problems. These problems are represented in toxic gases, as these gases lead to unknown diseases and affect the environment greatly (the phenomenon of global warming)^6^. In addition to these problems, the high cost of electricity generation and consumption causes more problems with the increase in energy demand, especially at peak electricity consumption, which has led many governments to resort to importing electricity, which is an undesirable matter because the external debt increases. The latter has become a major problem for some countries, which has forced these countries to resort to other more efficient solutions. Renewable resources are among the most prominent solutions that replaced the use of traditional resources in recent years because of their free and easy access to them, as the use of these resources leads to great protection of the environment compared to traditional sources^7^. Renewable energy sources, unlike traditional energy sources, are available throughout the world and are available throughout the year without cost, which makes their use unconditional, as their use in generating energy reduces the costs of producing and transmitting electrical energy and helps distribute it. The use of renewable sources does not lead to the release of toxic gases, which is of great importance for the environment. In addition, the use of these sources leads to a reduction in the bill of production and consumption of electric energy, which helps human and economic development in general^8^.

Wind power (WP)^9^, solar energy^10^, and water energy^11^ are among the most prominent power sources that are widely used at present around the world in the field of electric energy generation because of their ease of access and their permanent availability throughout the year. But WP is among these sources spread across land and sea because of the low cost of completion and the possibility of obtaining greater energies by developing wind farms (WFs) containing a large number of turbines designed to convert WP into electricity^12^. This technology in generating electrical energy depends on converting WP into mechanical energy and then generating electrical energy from mechanical energy, as it is necessary to generate sufficient energy from the wind for the purpose of obtaining electrical energy, which requires the use of turbines. The latter are mechanical converters that have the ability to convert WP into mechanical energy, as the size of this turbine is related to the amount of mechanical energy generated. Therefore, the integration of WP into the electric power generation infrastructure is of great importance and this is by harnessing the possibility of it working as an energy generator and at the same time as a system services provider, which makes WP at the forefront of sustainable and flexible energy solutions across the world in the future. The use of WP as an alternative solution in power generation is not limited to reducing toxic gases and protecting the environment, but rather its use leads to enhancing system stability and increasing the efficiency of energy networks significantly^13^.

In the future, the WP generation system will have a major role in the future energy generation scene and change the course of life, as the WP generation system is not limited to an active power (*Ps*) generator in the distribution network, but goes beyond much more than that. The generation system based on WP provides basic services similar to traditional power plants based on gas or coal, where in this system *Qs* is compensated and harmonic distortions are effectively mitigated, which helps to stabilize the energy generation system and increase its efficiency. Thus, wind turbines (WTs) contribute to a cleaner and more reliable electrical grid which is desirable^14^. Moreover, the use of WTs will significantly help reduce the cost of production, transmission, and distribution of electrical energy compared to traditional sources. Also, reducing the electrical energy consumption bill and thus eliminating all problems found in traditional systems.

Traditionally, WTs are the component responsible for converting WP into mechanical power, where the power gained is largely related to the size of the turbine^13,14^. These systems rely heavily on energy gained from the wind to generate electrical energy, as in order to obtain greater electrical energy, a turbine with large dimensions must be used, which makes it difficult to achieve on the ground. Also, giant turbines are characterized by complex technology, which increases costs, which is undesirable and increases the cost of energy production and consumption. Therefore, a more effective solution was thought of to overcome this problem and reduce costs.

In recent years, a new turbine has appeared under the name of the multi-rotor WT^15,16^, where several WTs of different capacities are used to form one turbine to increase the energy gained from the wind and increase the stability of the system. This new turbine has several advantages compared to traditional turbines that make it the appropriate solution, as its use reduces the size of the turbines and the costs of constructing the turbines, and this helps greatly in reducing the costs of producing electrical energy. In addition to reducing the area of WFs, which is desirable. As is known, wind farms occupy a large area of land, which increases costs, which is undesirable. But the use of this new technology for turbines will lead to a revolution in the production of electrical energy, as this technology is in continuous development despite its modernity. On the other hand, this technology is expensive, complex, difficult to control, and contains a large number of mechanical components compared to traditional WTs, which makes periodic maintenance very expensive^17^. However, these WTs provide 20% to 30% more energy than conventional turbines, which is a good percentage^18,19^. On the other hand, in the case of variable wind speed (WS), the doubly-fed induction generator (DFIG) is very suitable, as the rotational speed is controlled by feeding the rotor and thus controlling the amount of energy generated^20^. This generator is directly connected to the electrical grid from the static part without using any transformer, which reduces the cost of the generation system, which is an advantage not found in synchronous generators of all kinds. In addition, DFIG has several other advantages such as low cost, durability, low maintenance, ease of control, and its great ability to give good performance in the case of variable WSs. All these features make DFIG one of the most reliable solutions in the field of generating electrical energy from wind.

In the generation system that depends on the use of DFIG, two different inverters are used, the first task is to convert the alternating voltage into a continuous voltage and it is called the grid side converter (GSC) and the second task is to convert the output voltage of the GSC into an alternating voltage less than the grid voltage to feed the moving part of the machine It is called rotor side converter (RSC)^21^. These two inverters need a control strategy, as the control strategy chosen to control the inverters plays a big role in the quality of the power sent to the grid. In addition, a separate control inverter is used for each inverter, so the two inverters can use the same type of command (two similar strategies) or each inverter has its own command (two different strategies). Simplicity of control, durability, ease of implementation, and cost are almost the most prominent challenges for any reliable energy system for generating electrical energy, as these characteristics play an important role in implementing the energy system and thus in reducing the bill for production and consumption of electrical energy. Therefore, it is necessary to pay attention to these features before implementing any energy system. In addition, the number of gains of the control strategy is one of the most prominent factors through which the system’s response can be adjusted and good results can be obtained, as the fewer the number of gains, the easier it is to use smart strategies to determine the optimal values for these gains.

As it is known, there are many different control strategies, and each strategy has characteristics and advantages that make it suitable for some applications and not suitable for other applications. The most famous of these commands can be mentioned as direct power command (DPC)^22^, vector command (VC)^23^, field-oriented command (FOC)^24^, predictive command (PC)^25^, sliding mode command (SMC)^25^, direct torque command (DTC)^26^, passivity command^27^, high-order SMC^28^, synergetic command(SC)^29^, fractional-order command^30^, intelligent command^31,32^, hybrid command^33,34^, and backstepping command (BC)^35^. All of these mentioned strategies were applied to DFIG, where some strategies showed high performance such as BC technique and PC technique in improving the characteristics of the WP generation system such as current quality and robustness compared to some strategies such as FOC technique and DTC technique. However, these strategies have a defect represented in the complexity and difficulty of implementation, which makes the cost of the system rise, and this is inacceptable. In addition, these techniques are related to the system parameters, which creates several problems in the event of a malfunctions in the machine, which is not desirable at all. Techniques such as DTC technique and DPC technique are among the best controls that can be relied upon in the field of renewable energies because of their simplicity, ease of implementation, dynamic speed, and low cost compared to several techniques such as BC technique and passivity controls. These strategies rely on using a switching table (ST) to generate the pulses needed and two hysteresis comparators (HCs) to operate the inverter and control the characteristic quantities (such as torque, power, and flux). So, in these strategies, there are no gains that can be changed, and this is an advantage that makes these strategies a reliable solution. However, these strategies are characterized by several drawbacks that limit their spread despite the simplicity and ease of implementation that characterize them. These drawbacks can be identified in energy ripples, low current quality, and durability. Power quality and current are two of the most prominent problems in power systems that rely on turbines. These drawbacks can be attributed to the use of discrete value estimation, where the estimation process is related to the machine parameters (resistance *Rs*), which creates time delay problems and this is undesirable^35^. DTC technique and DPC technique offer high performance and good robustness compared to VC technique and FOC technique. The latter two rely heavily on the use of a proportional-integral (PI) regulator to command the *Ps* and *Qs* of the DFIG, which makes them less efficient in case of system parameters change. In addition, pulse width modulation (PWM) technique is used in these strategies to produce the necessary control pulses for the inverter, which creates high frequencies at the output of the inverter, and this may cause problems in the machine^36^. Also, the negative of these strategies (VC and FOC) lies in their reliance on knowing the mathematical model of the machine accurately, which makes it greatly affected in the event of a malfunction in the system, which is not desirable. The use of a mathematical model makes these strategies complex and difficult to implement in the case of complex systems. Therefore, it is necessary to search for other strategies with high performance, great durability, high efficiency, and great effectiveness in overcoming defects and problems.

Artificial intelligence (AI) is one of the most prominent topics of the hour because of its ability to overcome several problems and compensate for traditional controls, as AI strategies are considered one of the most prominent solutions in the field of renewable powers because of their high durability, its ability to operate in difficult conditions, easy application, and does not consume as much energy as it is. Most AI strategies depend on experience in application, as they do not need to know the exact mathematical model of the system under study, which makes them very suitable for any system. Moreover, it does not require a specialist or require great effort or costs, as it only requires knowing the number of entrances or exits. These strategies can be used to determine gain values, which is a good thing that helps improve dynamic response and overcome defects and problems. The case is in conventional strategies, which are strategies that are not related to the system, which gives it the ability to improve the characteristics of the system in the event of a defect^37^. AI strategies are diverse, where the most famous are neural networks (NNs)^38^, genetic algorithms^39^, fuzzy logic^40^, grey wolf optimization^41^, ant-colony optimization^42^, particle swarm optimization^43^, neuro-fuzzy algorithm^44^, etc. These smart strategies were used to overcome the shortcomings of several techniques such as DPC technique^45^, DTC technique^46^, VC technique^47^, SMC technique^48,49^, and third-order SMC technique^50^ of DFIGs. The use of smart strategies in these aforementioned works has greatly improved the characteristics of the systems and strategies compared to traditional strategies, as in most of these works, traditional controllers are replaced with smart controllers with distinctive performance and high accuracy. For example, in the DTC technique and DPC technique, the switching table was compensated by the NN technique in order to generate the pulses needed to operate the inverter^46,51^, where the use of this smart strategy led to a significant reduction of the *Ps* and *Qs* ripples while increasing the quality of the current compared to the conventional controls. In addition, it is noticed that the system has become more durable in the event of a malfunctions in the machine, which is a very good thing. One of the solutions that gave a good effect in the case of changing the system parameters is the hybrid controls, which is the combination of controls that are different or similar in principle and characteristics. One of the most prominent examples of combining controls can be found with the combination of BC technique and SMC technique to control DFIG^52^, where the resulting strategy (BC-SMC) is characterized by complexity and difficulty of implementation, especially in large systems. However, it provided excellent results in terms of improving system characteristics in the event of a system malfunctions compared to the DPC technique. This strategy uses capabilities estimation, as it uses the same estimation equations found in the DPC strategy. The results obtained from the MATLAB environment showed the superiority of the proposed strategy over the traditional strategy in terms of response time, power ripples, current quality, and robustness. However, the problem of energy ripples remains, which is undesirable and requires the use of a filter with the proposed system, which increases costs and this is undesirable. Instead, the strategy resulting from the combination of STA and SC techniques^53^ is simple, uncomplicated, highly efficient, robust, and can be easily accomplished compared to many other strategies. The SC-STA controller was used to overcome the problems and drawbacks of the DPC technique of DFIG strategy and power control. In addition to using the SC-STA controller, the PWM strategy was used to control the inverter and reduce the degree of system complexity. The DPC-SC-STA strategy was implemented in a MATLAB environment using a 1.5 MW DFIG with variable WS for the purpose of studying the behavior compared to the traditional strategy. Simulation results showed the superiority of the DPC-SC-STA technique in terms of reducing power ripples and current quality compared to conventional DPC technique and several other strategies.

Another effective strategy was obtained by combining SMC technique and SC technique to reduce power ripples and increase the efficiency of DFIG^54^, where the resulting controller was used to overcome the defects of the DPC technique. The HCs were eliminated and replaced by two SC-SMC techniques to command the *Ps* and *Qs*. Moreover, PWM technique was used to command a two-level inverter. The advantage of this control is simplicity, robustness, and ease of implementation. This proposed strategy was implemented in order to overcome the problem of low power quality and durability in the DPC strategy, as the MATLAB environment was used for the purpose of implementing and verifying the validity and safety of this algorithm. Using different tests, the proposed strategy proved its worth in improving the system properties compared to DPC technique and some other controls. However, the problem of energy ripples remains as a result of the use of the filter capacitor, and this appears clearly in the case of changing the parameters of the machine, which causes new research to obtain more efficient solutions for DFIG control.

The PC strategy remains one of the types of nonlinear controls that gave very satisfactory results, as it is an algorithm that differs from many commands in principle, degree of complexity, simplicity, and robustness^55^. This strategy is based on a predictive model of the process, which predicts future outputs based on historical information about the process and expected future inputs. This strategy depends on the mathematical model of the carefully studied system, which makes it difficult to implement in the case of complex systems, which is undesirable. Its use gives good results and significantly improves the characteristics of industrial systems. Thus, this strategy emphasizes the function of the model, not the structure of the model^56^. This strategy has been used in many different fields^57–61^. It has been used since the eighties of the last century in process industries in chemical factories and oil refineries. In^58^, PC technique was used to command the DFIG power. PC technique was used to overcome power fluctuations and greatly increase the system's robustness. This strategy uses power estimation to calculate the power error. Compared to the DPC strategy, the PC strategy is more complex and requires knowledge of the mathematical model of the machine. The MATLAB environment was used to implement this strategy, with comparison to traditional control, where the results showed a high performance in improving the current quality compared to the conventional command. In^59^, a DPC technique based on the PC technique was used to command the shunt *Ps* filter to improve current quality and increase system robustness. In this work, the PC technique was used to overcome the shortcomings of the DPC strategy, as the two HCs and ST were eliminated and replaced with both the PC algorithm and SVM technique. Using the latter increases the degree of complexity and makes the system more expensive, which is undesirable. But using the SVM technique increases the quality of the stream significantly. In addition, in addition to using the SVM strategy, the phase-locked loop (PLL) strategy is used to increase the stability and robustness of the system. The negative side of using the PLL strategy is the increase in the complexity of the system, the difficulty of implementing it, and the increase in its cost, which is undesirable. The proposed command was implemented using MATLAB software and this is in the case of different tests, where the results showed superiority of DPC-PC technique compared to the DPC technique in terms of current quality. According to the work done in^60^, model PC (MPC) is a strategy in which the control actions computed by the cost function are reduced to a dynamic system constrained on a finite and receding horizon, where at each time step, the MPC regulator receives or estimates the current state for the factory. A comparison of MPC technique and PI control can be made to indicate the effectiveness of PC technique, as MPC technique takes less time to reach the set point under steady-state error (SSE) conditions, and the offsets are smaller compared to the PI controller. In most applications dealing with PC technique, MPC technique proves to be superior in performance compared to PI controller results^61^. In general, this control is an advanced process control strategy that is used to control the process while meeting a set of constraints, where MPC technique has a major advantage that makes it allow to improve the current lead time while keeping the future periods in the calculation and this is done by optimizing a limited time horizon, So it executes the current time period and then optimize again, recursively. PC technique is different from square linear regulators because it can anticipate future events and can take control actions accordingly, which is not present in PI controllers.

As is well known, torque ripples are undesirable in power generation systems, as they can cause malfunctions in the mechanical components of the system, leading to a reduction in the life of the power train and the system as a whole. Also, it increases the periodic maintenance and thus the industrial cost. In^62^, the author proposes a new DTC technique for eliminating torque ripples for DFIG by using a PC technique. The proposed DTC-PC strategy differs from the traditional strategy in terms of concept, complexity, simplicity, durability, ease of implementation, number of gains, cost, performance, and is related to the mathematical model of the machine. In the DTC-PC strategy, the ST and two traditional HCs are eliminated and replaced by SVM and PC algorithms. The DTC-PC strategy uses the same torque and flux estimation equations found in the traditional DTC strategy, where this estimation is used to calculate the error in torque and flux. Estimating torque and flux are among the most notable drawbacks of this proposed strategy, in addition to the complexity and use of a mathematical model of the machine. In addition, this strategy results in low losses compared to the DTC strategy. For this, the DTC-PC strategy directly operates with the best rotor voltage space vector to reduce torque ripple and tracks the specified rotor flux amplitude to reduce losses, with no current control chain. As confirmed by simulations and experiments, the PC technique allows large stator frequency variations as required by the optimal flux order for minimal losses, while ensuring efficient torque ripple compensation.

The conventional model-free predictive current command (MFPCC) strategy of DFIG introduces high ripples for constant power and current harmonics, which is undesirable. This control uses only eight fundamental voltage vectors for a two-level transformer, causing distortion of the rotor and stator currents of DFIGs under an imperfect electrical grid unless special measures are taken. In^63^, an idea is proposed to overcome the drawbacks of the MFPCC technique. In the suggested solution, an extended finite command set (with 20 voltage vectors instead of 8 as in the traditional finite-command set) is used to improve the SSE. Also, the stator reference current is calculated so that sinusoidal and balanced stator currents are obtained even under a non-ideal electrical grid. The proposed strategy to overcome the problems of the MFPCC technique is characterized by complexity, high cost, and difficulty of implementation compared to the traditional strategy. The proposed strategy for the MFPCC technique was implemented in a MATLAB environment using variable WS. To show the superiority of the proposed control, the simulated results were compared with each of the MFPCC technique and MPC technique, where the results confirmed its superiority and its great effectiveness in improving the properties of DFIG-based WT systems.

Another paper on PC technique^64^ presents a fixed reference frame implementation of an iterative PC technique module under ideal and non-ideal stator voltage conditions. In this work, MPC technique and redundant control techniques are combined to control the DFIG power without the need to use the PLL technique. Moreover, in this proposed strategy it is not necessary to know the harmonic content of the non-ideal stator voltage beforehand but in this case, given the nature of the distortions, the use of PLL technique is necessary to estimate the phase angle and magnitude of the voltage fundamental component. This implies a significant advantage over previous techniques because no special considerations are needed on the mathematical model of DFIG. The proposed strategy was first implemented in the MATLAB environment using variable WS, while extracting the necessary graphical results in order to compare them with the results of the traditional strategy. The graphical results obtained showed the high performance of the proposed strategy compared to the traditional strategy. On the other hand, the proposed strategy was implemented experimentally using dSPACE 1104 in ideal and non-ideal network conditions, where DFIG was operated under variable rotor speed. The experimental results obtained greatly confirm the validity of the simulation results. Moreover, the experimental results show that the proposed PC technique is superior to the classical MPC technique in terms of power ripples and current quality.

In the field of control, optimal operation and reliable control are essential to ensure high efficiency and high ability to follow the loads of the systems, especially power generation systems from WP. Optimum operation and high performance are difficult to achieve using traditional linear controllers such as PI control. This is because generation systems are non-linear and contain many uncertainties. In some cases, it leads to a deterioration in the performance of the DFIG-based generation system in unbalanced conditions on the power grid, which is undesirable and increases the economic bill. In^65^, the author proposes a new nonlinear modeling strategy for DFIG considering non-equilibrium electrical grid conditions and that the dynamics of DFIG are nonlinear. In this work, a nonlinear PC technique for DFIG power control is derived, in which the prediction is based on an input–output feedback linear (IOFL) scheme. Also, control is derived by optimizing an objective function that takes into account economic and tracking factors under realistic constraints. Using the IOFL strategy increases the complexity and cost of completion, and this is undesirable despite the results obtained. Complexity is one of the most undesirable negatives, as it helps increase energy consumption. The suggested strategy was implemented using MATLAB software, where the results showed that the proposed strategy effectively reduces the erosion of the generation units under normal electrical grid conditions compared to the classical technique. Also, the proposed controller can significantly reduce rotor overcurrent under unbalanced electrical grid conditions and thus improve the ability of grid-connected WTs to withstand grid voltage faults.

The work done in^66^ presents a new methodology for primary frequency response (PFR) in a micro-grid through Finite-Control-Set Model PC technique (FCS-MPC) plus dangling control applied to GSC for DFIG. The FCS-MPC strategy proposed in this paper is a new strategy, different from the PC strategy, which increases the durability and significantly reduces power ripples, overcoming the problems and disadvantages of the PC strategy. This proposed strategy has many advantages, such as high performance and efficiency in improving performance, increasing durability, and overcoming the problem of changing system parameters. In this work, the RSC is responsible for keeping the WT running at its maximum power point (MPP) extraction in the event of a disturbance, while the GSC is responsible for processing the power required to re-establish the fine-frequency grid at its rated value. In addition, energy is stored in the battery storage system (BESS) connected to the DC link, the value of which is determined via FSC-MPC technique by continuously adjusting the value of the droop gain. The proposed command has significant benefits such as continuous operation when MPP is extracted, energy injection proportional to frequency imbalance, ability to impose constraints through command, and it does not use any type of communication between the storage and control system. FCS-MPC technique aims to limit the gain of the dangling controller, which increases the power needed to control the frequency of the fine grid. The simulation is used to verify the performance of the designed technique for an unexpected island of the small grid under different WS scenarios, where the simulation shows that the DFIG equipped with the designed technique can provide auxiliary services such as PFR in all different operating modes. In^67^, an analytical method for designing weight matrices based on a pseudo-algebraic Riccati equation named Fake Algebraic Riccati Equation (FARE) is proposed to ensure the stability of the DFIG-based control system. The simulation results are used to choose the required control horizon and the desired prediction horizon, taking into account the SSE and computational cost. Moreover, the designed technique of the DFIG vector control model, the MPC theory and the FARE formula were verified by simulation and experiment. The experimental results validate the simulation results, confirming the superiority of the proposed strategy in terms of improving the DFIG properties compared to the traditional strategy. Work^68^ proposes a low-complexity model strategy based on direct predictive power command (LC-MPDPC) for DFIG under balanced and unbalanced electrical grid conditions. Appropriate formulations of the LC-MPDPC strategy are obtained by using the detailed analysis of the mathematical model of DFIG, in which a unified energy compensation strategy is proposed to flexibly achieve different control objectives during the unbalanced network condition. The LC-MPDPC strategy differs from the DPC strategy and some controls, as the ST and two HCs are not used in this proposed strategy. Also, the LC-MPDPC strategy relies on capacity estimation like the traditional strategy, which makes it affected in the event of a malfunction in the system. Compared to the DPC strategy, the LC-MPDPC strategy is characterized by a greater degree of complexity, difficult to implement, expensive, related to the mathematical model of the generator, and the presence of a significant number of gains. However, this strategy has outstanding performance in reducing power ripples and increasing the quality of the current. Also, it has great robustness compared to the traditional strategy. The designed technique was implemented experimentally using dSPACE 1104 card, where the obtained experimental results were compared with the DPC technique results under the balanced network condition. Experimental results prove the superiority of the LC-MPDPC technique over DPC technique for the DFIG system under the condition of an unbalanced network with the satisfactory dynamic response when the control target changes. In^69^, the PC technique of the finite-control-set model-predictive command (FCS-MPC) for the DFIG system is presented, in which the switch states of the GSC are directly taken as control inputs. Therefore, the improved control procedure can be applied directly to the GSC. The proposed control has a salient feature that makes it better than current FCS-MPC approaches, as this feature is reduced computation time. In the proposed control, a set of enhanced decision variables is introduced and the original FCS-MPC technique intractable binary quadratic programming problem can be transformed into a binary linear programming problem that can be solved efficiently. Thus, the calculation time of the proposed scheme is much less than the time of other strategies schemes, as this reduction in calculation time leads to better control performance with longer forecast horizons.

In recent years, advancements in WF technologies have become pivotal in addressing the challenges associated with renewable energy integration into the existing power grid. A significant contribution to this field is found. In^70^, which presents a comprehensive analysis of WF power dispatching, emphasizing local power management and controller units. The authors propose a novel local power management algorithm for *Qs* production in individual wind generators. This algorithm considers three operational modes of the WF and incorporates the maximum *Qs* of the GSC and the DFIG. It relied on conventional control based on the PI controller. In^71^, the integration of WFs into the power grid is addressed, focusing on the efficient management of *Ps* and *Qs*. Utilizing squirrel cage induction generators and back-to-back converters, the research employs a proportional distribution algorithm for optimal power allocation to individual WTs. The study emphasizes the development of local power management and control units for WTs to extract maximum power from the wind and meet predetermined requirements. The proposed strategies ensure the safe integration of WFs into the electrical grid, respecting grid code requirements and power system stability. In^72^, a critical examination of a WF with DFIGs during partial load operation is presented. Two equivalent models are compared to a detailed model, focusing on different aggregation methods. The second model, which aggregates only the electrical systems for each WT group (WTG), closely mirrors the detailed model's response. The study applies power control and management to this second aggregated model, utilizing a PI controller for regulating *Ps* and *Qs*., proving effective across three operating modes. In addressing^73^ demonstrated significant advancements. Their study, 'Grid synchronization of WFs with DFIGs using nonlinear integral BC technique,' employs a semi-aggregation method to obtain an equivalent WF model. The main focus is on achieving effective synchronization between the WF and the electrical grid by controlling the rotor currents of DFIGs. Through a comparative study with a PI controller, the nonlinear integral BC (IBSC) algorithm proves superior, offering enhanced efficiency and performance. Addressing the challenges posed by increased WP integration, Atallah et al.^74^ introduces a supervisory control strategy. Focused on a 50 MW WF with pitch-controlled turbines, the strategy enables *Ps* and *Qs*. control, incorporating automatic voltage and frequency control with droop and dead band. The study evaluates the strategy's performance against Danish TSO requirements, highlighting its potential in enhancing grid stability during the rise of WP penetration. In 'supervision and operation of a large WF connected to the electrical grid,' Atallah et al.^75^ introduces an innovative system for managing a WF connection to the electrical grid. The proposed algorithm optimally distributes *Ps* and *Qs* references to individual turbines based on aerodynamic power and WS. Using vector-FOC (PI-conventional) and a five-level neutral point-clamped converter, the system demonstrates effective closed-loop control, maintaining stable DC voltage and adapting to the electrical grid with enhanced waveform resolution. In 'Enhancing Frequency Stability in Power Systems'^76^, an adaptive deloading scheme for variable-speed WT generators is introduced. This approach strategically utilizes deloading to create a power reserve, improving primary frequency response during system contingencies. Using a Lagrange interpolating polynomial on the turbine's nonlinear power curve, the scheme dynamically regulates output power, outperforming traditional controls in scenarios like WS fluctuations. Real-time hardware-in-the-loop simulations validate the method's effectiveness, making it promising for wind-integrated power systems and grid code compliance.

From the literature, it is evident that PC technique is widely recognized for its robustness and performance. In the context of WFs, their significant impact on supporting the electrical grid in terms of energy and addressing various electrical grid scenarios is apparent. However, the existing literature has not extensively explored the role of a WF as a crucial element in filtering when connected to the electrical grid with non-linear loads. The emphasis on power quality and performance improvement in this regard is relatively limited.

In this paper, the use of finite space model predictive command (FS-MPC) is proposed to manage and control a WF for the production of electric power, where the DFIG is used for power generation. This farm consists of four WTs that together generate a total power of 6 MW. Accordingly, the main contribution of the paper lies in the management and regulation of WF operations using FS-MPC control, and effective handling of non-linear load integration. This completed study is of great importance in the industrial field, as it gives a complete picture of how to command the WF and its management using the PC technique. The latter, its use gives high performance and efficiency in farm management compared to classical technique. MATLAB software was used to implement and verify the WF management using four 1.5 MW DFIG generators. Among the objectives achieved are the following:

- Implementation of a proposed surveillance algorithm for the efficient management of WF which for improving its performance has four operational modes based on PC technique. This strategy works on fault mode which depends on fault ride-through technology, with the primary goal of protecting the WF from external influences stemming from the electrical grid (voltage drop).
- Introducing a novel algorithm designed to assist the WF in filtering the electrical grid when connected to non-linear loads. This algorithm focuses on effectively managing the filtering process and incorporating a controlled number of WTs based on the current absorbed by these loads, Tests are performed on the proposed algorithm using various types of nonlinear loads to validate the effectiveness of the novel algorithm.
- Minimizing power ripples compared to classical control.
- Reducing current distortions significantly.
- Increasing the robustness of the WF.

The constituent sections of the paper are: The first section is a comprehensive introduction to the topic covered in this paper. The second section presents an overview of the proposed farm with the proposed control for the management and organization of this WF. In the third section, the necessary simulation results are given with a comparison of the results with the classical technique. Finally, a summary of the works carried out and the mention of future works are presented.

## Proposed WF and control

**Figure 1:**
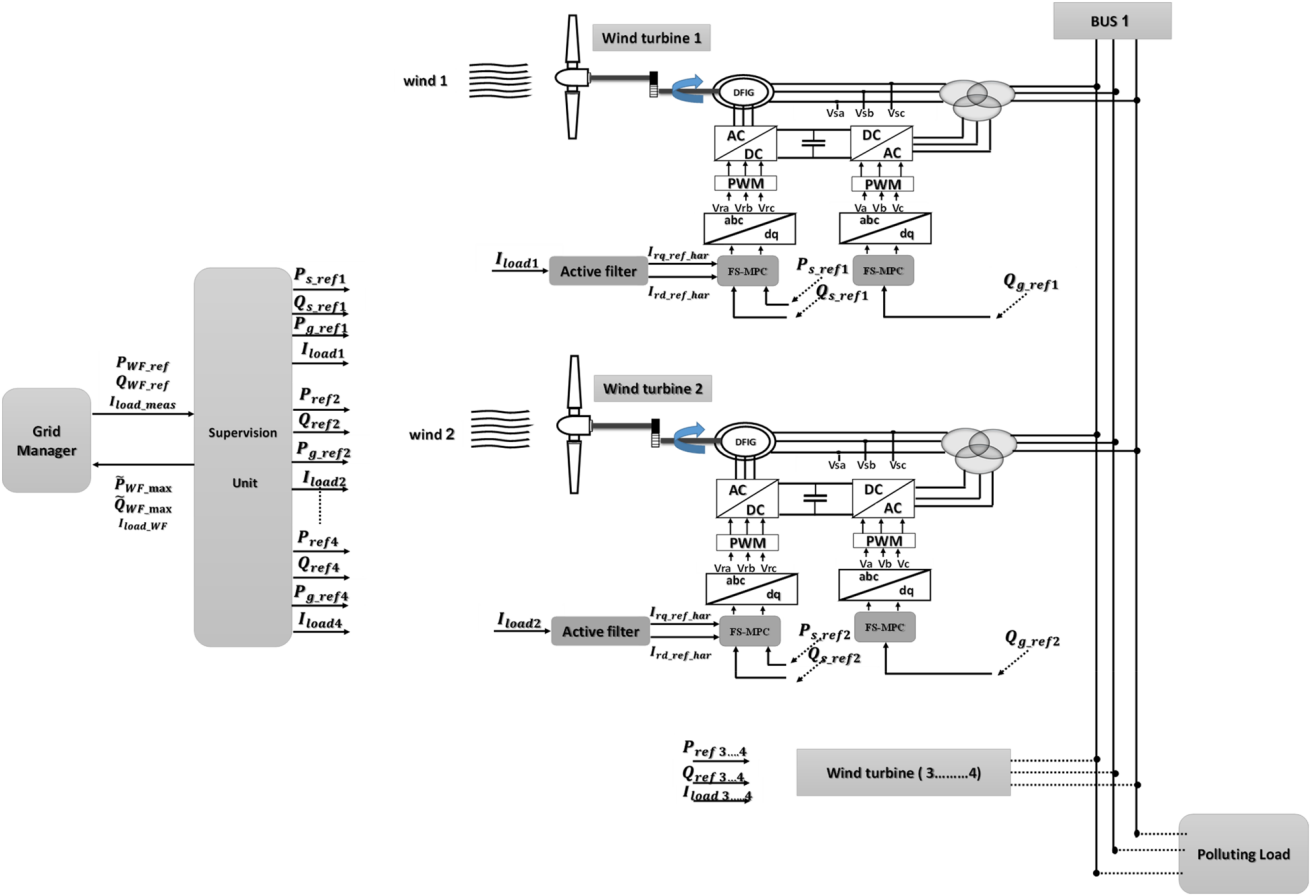
Block diagram of the studied system.

In this section, a new energy system is proposed that relies on the use of a group of turbines to generate electrical energy from wind using a new nonlinearity control algorithm to manage the proposed system. This completed research differs from many scientific works in terms of the idea, principle, results, degree of complexity of the system, control strategy, and the results obtained. This proposed work is of great importance in the field of renewable energies, as it gives a new idea about filtration in the field of wind energy. So, a WF with a total power of 6 MW comprising four WTs is analyzed. Additionally, the WF is equipped with four selective filters, as visually represented in Fig. 1. To manage and regulate the farm's operations, it is governed by the FS-MPC technique, effectively handling the integration of a non-linear load.

The WF is based on the DFIG model, as detailed in^77^.

The DFIG is represented in the d-q frame, which is associated with the rotating field. This modelling approach is employed to streamline the representation of the DFIG. Specifically, the stator flow direction on the d-axis of the Park frame is utilised for simplification purposes. In this context, it is assumed that the wind turbine is connected to a stable electrical grid. Additionally, for the sake of simplicity in the model, the voltage drop across the stator resistance is disregarded.

### Algorithm based on the proportional distribution of power references

The algorithm based on the proportional distribution was developed to distribute the power instructions in a proportional way on the WTs of the farm. The algorithm was divided into central and local supervision units. This algorithm aims to manage the proposed energy system well to avoid any possible defect or problem occurring on the farm.

#### Central supervision unit of the WF

The algorithm utilizing proportional distribution has been devised to efficiently allocate power instructions across the WTs within the farm^78^. With a strong emphasis on safety, this algorithm guarantees that each WT consistently operates within the boundaries specified by the (*P* and *Q*) diagram in^70^. As is known, it is necessary to manage energy well in a wind farm in order to obtain distinctive performance of the turbines and produce sufficient energy from the wind. Therefore, choosing an algorithm characterized by distinctive and high performance gives good results and reduces the possibility of problems occurring on the WF.

The primary objective of this algorithm is to determine the *Ps* and *Qs* references for each WT, denoted as $P_{{_{WG\_i} }}^{ref}$ and $Q_{{_{WG\_i} }}^{ref}$, respectively, based on the total *Ps* and *Qs* references requested by the grid manager, represented as $P_{WF}^{ref}$ and $Q_{WF}^{ref}$^75^. To assess the *Ps* production power of the farm, the algorithm aggregates the maximum *Ps* available at each WTs level within the entire farm^70^. This ensures an effective and balanced distribution of power among the turbines, optimizing their performance while adhering to the electrical grid manager's requirements.

1 $$\mathop P\limits^{ \sim }{_{WF\_max}} = \sum\limits_{i = 1}^{n} {\mathop P\limits^{ \sim }{_{WG\_max\_i}} }$$

Similarly, the *Qs* production capacity of the farm is estimated by summing all the maximum *Qs* that each WT on the farm can produce.

2 $$\mathop Q\limits^{ \sim }{_{WF\_max}} = \sum\limits_{i = 1}^{n} {\mathop Q\limits^{ \sim }{_{WG\_max\_i}} }$$

$\mathop P\limits^{ \sim }{_{WG\_max\_i}}$ and $\mathop Q\limits^{ \sim }{_{WG\_max\_i}}$ are the *Ps* and *Qs* of the WT $i$.

$\mathop P\limits^{ \sim }{_{WF\_max}}$ and $\mathop Q\limits^{ \sim }{_{WF\_max}}$ are the total *Ps* and *Qs* of the WF.

$n$ represents the number of WTs on the WF.

The allocation of *Ps* and *Qs* references ($P_{{_{WG\_i} }}^{ref}$, $Q_{{_{WG\_i} }}^{ref}$) follows a proportional distribution approach. Each WT contributes a portion of its maximum *Ps* and *Qs*. This strategy guarantees that the WT with the highest *Ps* production capacity will receive the highest *Ps* reference. Similarly, the WT with the greatest capacity for *Qs* production or consumption will play a more significant role in managing the *Qs* of the entire WF^70^. The proportional distribution mechanism ensures an equitable and optimized utilization of resources, enabling efficient power management across the WTs in the farm.

3 $$P_{WG\_i}^{ref} = \frac{{\mathop P\limits^{ \sim }{_{WG\_\max \_i}} }}{{\mathop P\limits^{ \sim }{_{WF\_\max} } }}P_{WF}^{ref}$$

4 $$Q_{WG\_i}^{ref} = \frac{{\mathop Q\limits^{ \sim }{_{WG\_\max \_i}} }}{{\mathop Q\limits^{ \sim }{_{WF\_\max} } }}Q_{WF}^{ref}$$

The key benefit of employing this strategy lies in its ability to ensure a safe operational margin for all WTs on the farm^79^. Protecting the turbine from risks is necessary, which reduces regular maintenance costs and the risk of the turbine being damaged. Also, it is necessary to maintain energy production within normal limits and regularly in order to supply the network with the necessary energy. By maintaining a sufficient bound from their maximum production capacities, the risk of WT saturation is effectively mitigated^80^. Even if one turbine reaches saturation, operating at its maximum production or consumption, the excess power can be seamlessly transferred to other WTs still capable of meeting the demand^78^. However, implementing this strategy involves a certain level of complexity, as it necessitates information on the available aerodynamic power of all WTs. The challenge lies in accurately estimating this power at each turbine level, which directly depends on the WS-a parameter that can be challenging to measure and, consequently, introduces some approximation in the process^75^.

**Figure 2:**
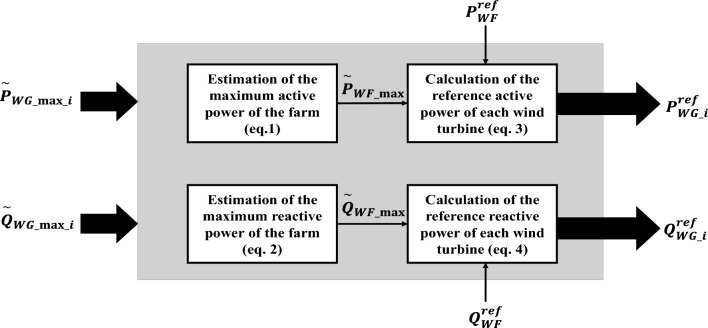
WF power management: utilizing a proportional distribution approach.

Figure 2 presents a schematic representation of the algorithm discussed in this paper. The diagram illustrates the key steps and components of the algorithm, providing a visual overview of its operation.

#### Local supervision unit of the WF

The total produced power (*P*_*WG_i*_ and *Q*_*WG_i*_) is divided into two components: stator power (*P*_*s_i*_ and *Q*_*s_i*_) and GSC power (*P*_*g_i*_ and *Q*_*g_i*_). To ensure a seamless power management process between the GSC (*P*_*g_ref_i*_ and *Q*_*g_ref_i*_) and the DFIG stator (*P*_*s_ref_i*_ and *Q*_*s_ref_i*_), each wind generator relies on its local controller unit. This local controller unit coordinates the distribution of *Ps* and *Qs* references (*P*_*WG_ref_i*_ and *Q*_*WG_ref_i*_) as provided by the central dispatching unit. Through this coordination, the wind generators optimize their power contributions and maintain efficient operation within the overall system.

Maintaining precise control over the WF *Ps* is of paramount importance. Therefore, it is necessary to choose a control strategy with high characteristics such as robustness, outstanding performance, and efficiency in improving system characteristics. Also, the proposed strategy must be characterized by simplicity, quick dynamic response, and ease of implementation in order to reduce the complexity and cost of the system. All of these features help significantly reduce the electricity production and consumption bill. This meticulous management not only ensures a more efficient utilization of the wind generators but also guarantees the safe and reliable operation of the electrical systems. To determine the *Ps* references for both the DFIG stator and the GSC, the losses in power electronic converters and filters are disregarded, resulting in the following expressions^73^:

5 $$P_{s\_ref\_i} = P_{WG\_ref\_i} - P_{r\_i}$$

6 $$P_{g\_ref\_i} = P_{r\_i} - P_{dc\_ref}$$

7 $$P_{dc\_ref} = i_{DC\_ref} V_{DC}$$

where,$P_{r\_i}$ represents the rotor *Ps*; $P_{dc\_ref}$ denotes the power required for DC bus voltage control; $i_{DC\_ref}$ signifies the DC current reference derived from a PI controller utilized for DC voltage control.

The coordination of *Qs* distribution between the DFIG stator circuit and the GSC is effectively achieved through the proposed *Qs* management algorithm. This algorithm serves to determine the appropriate *Qs* references for both the DFIG stator circuit and the GSC (*Qs*_*_ref*_ and *Q*_*g_ref*_)^73^. The management of *Qs* encompasses four distinct modes, each corresponding to specific operating points dependent on the turbine speed: Maximum power point tracking (MPPT) mode, delta control mode, Fault mode, and filtering mode.

The algorithm's optimal performance is ensured by adhering to the maximum power constraints of both the GSC and DFIG stator circuits. As a result, the calculated *Qs* references for the DFIG stator must be constrained by their respective maximum *Qs* capabilities^70^. The maximum *Qs* of the DFIG ($Q_{s\_\max }$) is determined through the utilization of the rated stator and rotor currents, rated rotor voltage, and the steady-state stability limit^80^. Similarly, the maximum *Qs* of the GSC ($Q_{g\_\max }$) is computed using its PQ diagram. This determination primarily considers factors such as the GSC's DC link voltage, employed modulation technique, rated current of the GSC's power electronic components, and the converter size. Through these computations, the algorithm ensures an effective and efficient management of *Qs*, maintaining stable and optimized operation across the WF system^70^.

##### Mode 1 (MPPT)

During periods of low WSs, the wind generator operates in the maximum power point tracking (MPPT) mode. However, the conventional wind system often lacks the capacity to compensate for the necessary *Qs* demand in this mode. This limitation arises because the conventional system directs its power production capabilities toward maximizing *Ps* generation through the DFIG's stator. In contrast, when employing the recommended algorithm within a wind system, the DFIG's stator operates with the freedom to contribute the entire *Ps* output. Simultaneously, the GSC takes on the responsibility of furnishing the requisite *Qs* within its predefined maximum threshold.

Should the *Qs* reference ($Q_{WG\_ref\_i}$) surpass the GSC's maximum *Qs*, the GSC steps in to deliver its maximum *Qs* output. The DFIG's stator then supplements the power generation by providing the remaining portion of the required *Qs*. This coordinated approach ensures a balanced distribution of *Ps* and *Qs* while maintaining operational stability within the defined limits of the system.

##### Mode 2 (delta)

During instances of elevated WSs, the WF transitions to the delta control mode, deliberately curbing its *Ps* output to a level below its maximum capacity $0 < P_{WF\_ref} < P_{WF\_\max }$. In this scenario, collaborative efforts between the GSC and the DFIG stator come into play, jointly addressing the complete demand for *Qs*.

To realize this, the reference *Qs* for both the GSC ($Q_{g\_ref\_i}$) and the DFIG stator ($Q_{s\_ref\_i}$) are computed using the proportional distribution technique. This approach ensures that the *Qs* requirements are shared appropriately between these two components. Through this orchestrated interplay, the WF maintains its *Ps* within controlled parameters while effectively managing the *Qs* demand, ensuring the harmonious operation of the system during higher WSs.

##### Mode 3 (fault)

This control mode becomes operational in the event of an electrical grid fault, such as a voltage dip. During such occurrences, the supervisory unit assumes a critical role in control. Activation of this mode is contingent upon the estimated voltage drop magnitude and the reliability of the control system in use.

Knowing, the acceptable range for FRT (Fault Ride Through) deviation is within limits such as ± 5% or ± 10% of the rated voltage value. These limits ensure that the voltage levels in the distribution system remain within a specified tolerance to maintain the stability and reliability of the electrical supply. Adhering to these standards helps to ensure the proper functioning of connected equipment and devices, as well as compliance with safety and performance requirements set by relevant authorities.

**Figure 3:**
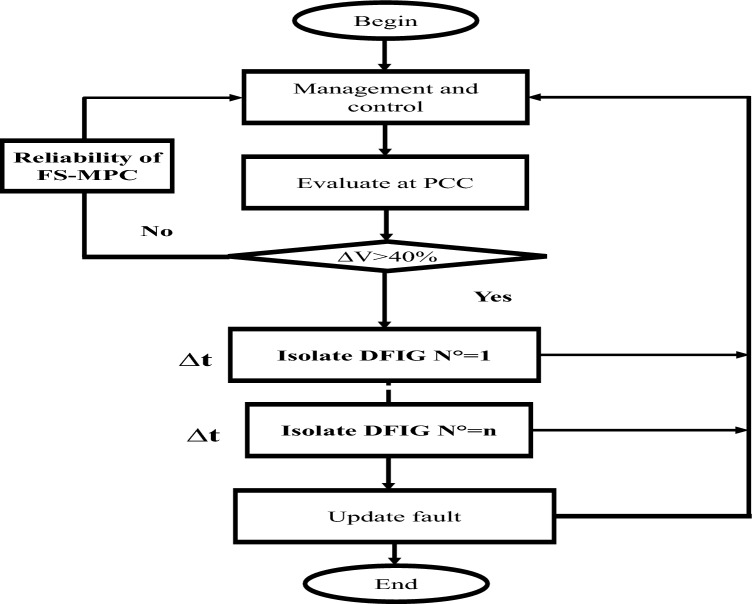
The proposed algorithm for a voltage drop protection system.

When the voltage drops falls within the range of 10–20%, the FS-MPC control possesses the capability to mitigate this voltage drop. However, for drops exceeding 20% up to 100%, this mode is triggered to safeguard the WF against the sudden peak, which can significantly impact the farm units. This process adheres to an algorithm delineated in Fig. 3. This coordinated response ensures the stability and dependability of the system during fault situations, thereby upholding the continued WF operation and the connected electrical grid at Drop% < 20%, and protecting the WF at Drop% > 20%.

##### Mode 4 (filtering)

In our paper, we introduced a novel operational mode designed to consolidate three distinct modes (MPPT, Delta, and Fault) into a single comprehensive approach. The primary objective of this mode is to ensure real-time validity for these different operating modes while considering the current state of the electrical grid. This approach becomes particularly relevant in situations where the electrical grid includes non-linear loads with significant current capacities. In such scenarios, the proposed mode enables the monitoring and control of the electrical network to effectively handle these complex loads. The mode incorporates a filtration process that utilizes the proportionality between farm units, allowing for efficient management of filtering. By adopting this approach, the WF can maintain stable and reliable operation while optimizing power utilization and enhancing the overall performance of the interconnected electrical grid.

### Filtering system offered by FS-MPC technique

**Figure 4:**
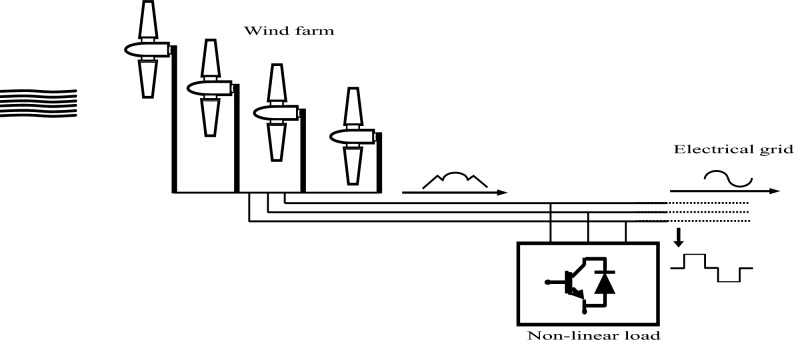
A novel model employing the wind farm as a filtering mechanism.

The main objective of this research is to introduce a novel model designed to mitigate electrical grid distortions arising from nonlinear loads, which, in turn, lead to mechanical fatigue in electric generators. The proposed solution involves employing the WF as a filtering mechanism (Fig. 4). Consequently, a software program is developed, offering the flexibility to select a specific number of DFIGs for filtering purposes. Depending on the maximum current intensity of the nonlinear load, users can opt for one, two, three, or all four DFIGs to perform the filtering process efficiently.

The operational management in this context encompasses six primary objectives, which are illustrated in Fig. 1:

- *Ps* and *Qs* reference comparison

The first objective involves transmitting the *Ps* and *Qs* reference values to each WT. This comparison is made between the reference power requested by the grid manager and the energy generated by the WF under the supervision unit.

- Power reference definition and FS-MPC technique

The third objective entails a two-step control process. Initially, a power reference is defined using the supervision of the local unit. Subsequently, the DFIGs-based WTs is controlled using an FS-MPC controller, which relies on the references provided by the first PI regulator.

- Program for DFIG activation based on load current intensity

To achieve the fourth objective, a program is developed, enabling the command to activate a certain number of DFIGs. The activation process is determined based on the intensity of the load current, facilitating the initiation of the filtering process.

- Selection of DFIG Units for Grid Filtering

The second objective revolves around the selection of a specific number of DFIG units responsible for filtering the electrical grid. This selection process is conducted within the framework of the supervision unit.

- Harmonic current references and rotor equivalents

In the pursuit of the fifth objective, the references of the harmonic currents are identified, and their corresponding rotor equivalents are defined.

- Control of grid currents for improved performance

The sixth objective centers on controlling the grid currents to ensure a superior THD rate and maintain a unit power factor, thereby enhancing the overall performance of the system.

#### Algorithm for the proposed approach

**Figure 5:**
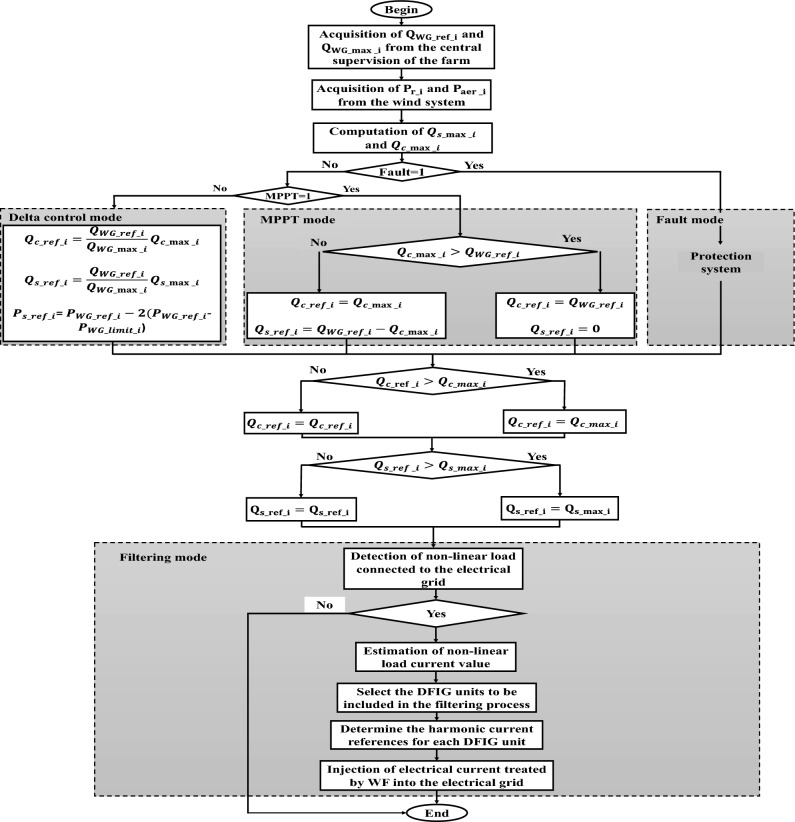
The proposed power supervision algorithm.

- *Step 1* The first step involves estimating the effective value of the current consumed by the nonlinear load. This load can introduce irregularities and distortions into the electrical current waveform.
- *Step 2* After estimating the load's current, the algorithm (Fig. 5) proceeds to determine the filtering power that each DFIG can provide. It's important to ensure that this filtering power remains within the predefined limits of each DFIG's capabilities.
- *Step 3* Using the filtering capacity, the algorithm (Fig. 5) then allocates the appropriate proportion of the estimated load current to each individual DFIG unit. This distribution is done in a way that maximizes the filtering effect and minimizes the impact of the nonlinear load on the system.
- *Step 4* Determine the harmonic current references for each DFIG unit identified in the process.
- *Step 5* Inject the treated electrical current from the filtration process into the electricity grid.
- *Step 6* Finally, the overarching goal of the algorithm is to achieve a sinusoidal waveform for the electrical grid. This is regardless of the presence of various harmonics introduced by the nonlinear load. By intelligently distributing the filtering task among the DFIG units, the algorithm works to maintain a clean and stable grid waveform, enhancing the overall performance and reliability of the system.

#### References harmonic generator

The selective filter employed in this system serves as a band-pass filter, specifically designed to isolate and effectively filter the current harmonic components present in the reference signal (α and β). We express the fundamental components $\hat{x}_{\alpha }$ and $\hat{x}_{\beta }$ as a function of $x_{\alpha }$ and $x_{\beta }$^81^:

8 $$\hat{x}_{\alpha } (s) = \frac{k}{s}\left( {x_{\alpha } (s) - \hat{x}_{\alpha } (s)} \right) - \frac{{w_{c} }}{s}\hat{x}_{\beta } (s)$$

9 $$\hat{x}_{\beta } (s) = \frac{k}{s}\left( {x_{\beta } (s) - \hat{x}_{\beta } (s)} \right) - \frac{{w_{c} }}{s}\hat{x}_{\alpha } (s)$$

**Figure 6:**
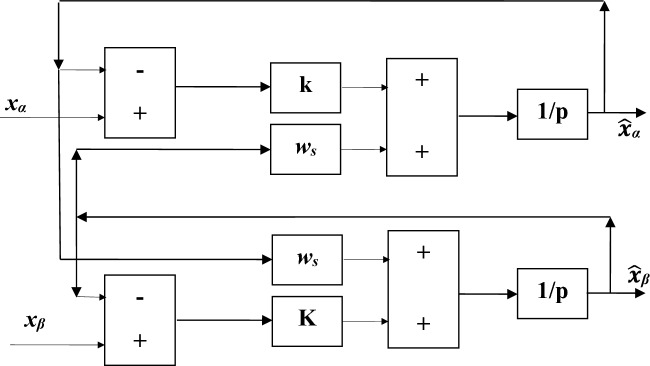
Diagram of the selective filter.

In the context of the reference signal (α and β), the components $x_{\alpha \beta }$ and $\hat{x}_{\alpha \beta }$ represent successive measurements of the signal and its fundamental component, respectively. Additionally, $k$ denotes a constant value, while $w_{c}$ corresponds to the pulse of the filtered signal. Furthermore, $s$ signifies the Laplace operator involved in the calculations. Figure 6 represents a diagram of the selective filter.

#### Model-based predictive current control

The foundation of the suggested PC strategy rests upon the concept that a static power converter can generate a finite set of potential switching states. The system's ability to forecast the variables' behavior for each of these states is key. Establishing the optimal switching state requires the definition of a selection criterion, which involves a cost function evaluating the anticipated values of controlled variables. Projections of these variables' future values are computed for every potential switching state. The state that yields the least cost, as determined by the cost function, is subsequently selected, culminating in an effective PC approach.

**Figure 7:**
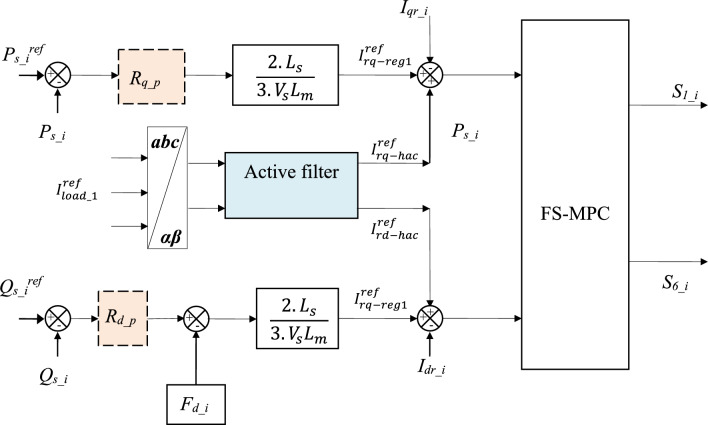
Block diagram of a proposed control structure.

To achieve the dual objectives of the regulator, it is essential to introduce two reference currents into the quadratic components of the rotor current (Fig. 7). The d-q rotor currents are defined as follows:

10 $$I_{rq\_i}^{ref} = i_{{rq_{ - } reg1\_i}}^{ref} + i_{{rq_{ - } har\_i}}^{ref}$$

11 $$I_{rd\_i}^{ref} = i_{{rd_{ - } reg1\_i}}^{ref} + i_{{rd_{ - } har\_i}}^{{\text{ref }}}$$

The designed PC technique uses the ability of a converter to create only a limited number of commutation states. The following formula can be used to describe the voltage equation of the rotor in the synchronous rotation condition^82^:

12 $$\left\{ {\begin{array}{*{20}l} {v_{rd\_i} = R_{r} i_{rd\_i} + \sigma L_{r} \frac{d}{dt}i_{rd\_i} - g_{i} \omega_{s} \sigma L_{r} i_{rq\_i} } \hfill \\ {v_{rq\_i} = R_{r} i_{rq\_i} + \sigma L_{r} \frac{d}{dt}i_{rq\_i} + g_{i} \omega_{s} \sigma L_{r} i_{rd\_i} + g_{i} \frac{{L_{m} V_{s} }}{{L_{s} }}} \hfill \\ \end{array} } \right.$$

Grid-side converter currents are synced with the grid voltage's Park reference frame. In this context, the filter (*Rg* and *Lg*) electrical equations may be simplified as follows:

13 $$\begin{gathered} V_{dg\_i} = R_{g} i_{dg\_i} + L_{g} \frac{{di_{dg\_i} }}{dt} - \omega_{s} L_{g} i_{qg\_i} + V_{g\_i} \hfill \\ V_{qg\_i} = R_{g} i_{qg\_i} + L_{g} \frac{{di_{qg\_i} }}{dt} - \omega_{s} L_{g} i_{dg\_i} \hfill \\ \end{gathered}$$

In FS-MPC technique, the discrete form of Eqs. (12) and (13), taking the basic “Euler” approach, can be represented as follows:

14 $$\left\{ {\begin{array}{*{20}c} {i_{dr\_i} \left( {k + 1} \right) = \left( {\frac{{T_{s} }}{{\sigma L_{r} }}(V_{dr\_i} \left( k \right) - R_{r} i_{dr\_i} (k) + g_{i} \omega_{s} \sigma L_{r} i_{qr\_i} (k)) + i_{dr\_i} \left( k \right)} \right)} \\ {i_{qr\_i} \left( {k + 1} \right) = \left( {\frac{{T_{s} }}{{\sigma L_{r} }}(V_{qr\_i} \left( k \right) - R_{r} i_{qr\_i} (k) - g_{i} \omega_{s} \sigma L_{r} i_{dr\_i} (k) - g_{i} \frac{{L_{m} V_{s} }}{{L_{s} }}) + i_{qr\_i} \left( k \right)} \right)} \\ \end{array} } \right.$$

15 $$\left\{ {\begin{array}{*{20}c} {i_{dg\_i} \left( {k + 1} \right) = \left( {\frac{{T_{s} }}{{L_{g} }}(V_{dg\_i} \left( k \right) - R_{g} i_{dg\_i} \left( k \right) + \omega_{s} L_{g} i_{qg\_i} \left( k \right) - V_{g\_i} ) + i_{dg\_i} \left( k \right)} \right)} \\ {i_{qg\_i} \left( {k + 1} \right) = \left( {\frac{{T_{s} }}{{L_{g} }}(V_{qg\_i} \left( k \right) - R_{g} i_{qg\_i} \left( k \right) + \omega_{s} L_{g} i_{dg\_i} \left( k \right)) + i_{qg\_i} \left( k \right)} \right)} \\ \end{array} } \right.$$

where, *Ts* is the sample period, *i*_*dr*_*(k), i*_*dr*_*(k), i*_*qr*_*(k), i*_*dg*_*(k),* and *i*_*qg*_*(k)* are the rotor and filter measured currents, respectively at *kTs. V*_*dr*_*(k), V*_*qr*_*(k), V*_*dg*_*(k,),* and *V*_*qg*_*(k)* are the rotor and filter voltages derived from the optimum voltage vector implemented in the (k)th sampling period.

The quality function ensures the good efficiency of the dynamic control. The cost function is calculated for each feasible switching state of the converter for each sampling period, choosing the one with the least error for the following sample period. Equation (25) defines the function of cost:

16 $$F_{j} = \left( {\Delta i_{dr\_i} \left( {k + 1} \right)^{2} + \Delta i_{qr\_i} \left( {k + 1} \right)^{2} + \Delta i_{dg\_i} \left( {k + 1} \right)^{2} + \Delta i_{qg\_i} \left( {k + 1} \right)^{2} + i_{m} } \right)$$

where$$i_{m} = \left\{ {\begin{array}{*{20}c} {\infty ,if\left| {i_{k + 1} } \right| > \left| {i_{\max } } \right|} \\ {0,otherwise} \\ \end{array} } \right.,\left| {i_{k + 1} } \right| = \sqrt {i_{d} \left( {k + 1} \right)^{2} + i_{q} \left( {k + 1} \right)^{2} }$$.

**Figure 8:**
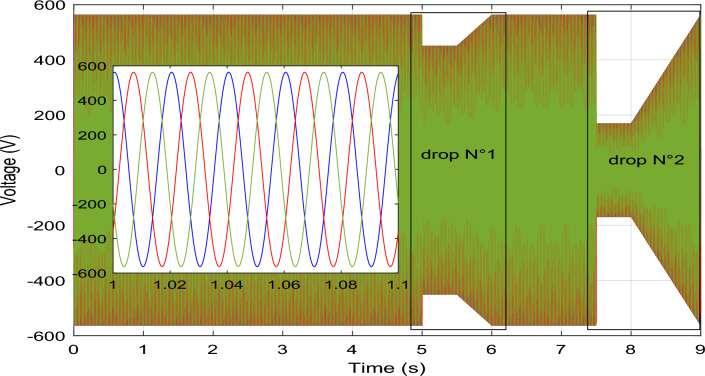
Grid voltage at PCC.

*i*_*m*_ represents DFIG and GSC overcurrent protection. The related voltage vector is disregarded if the current exceeds the limit value.

where R_q-p_ and R_d-p_ represent the *Ps* and *Qs* regulators, respectively (Fig. 8).

## Validation and discussion

**Figure 9:**
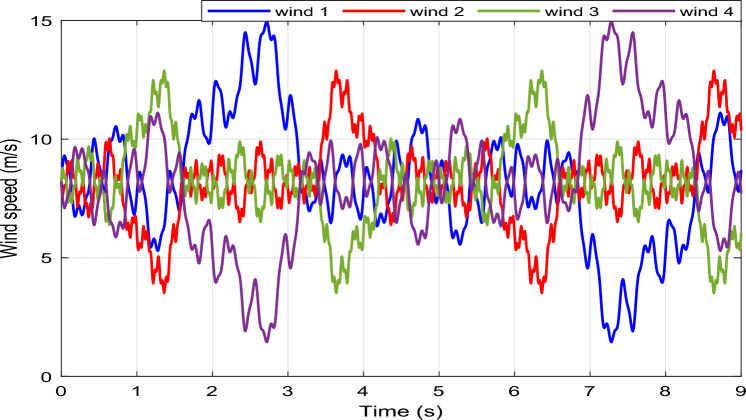
The four WSs.

This section of the research paper serves to confirm the validity and efficiency of the designed management technique. The simulation of WF operations and control is conducted using the MATLAB/Simulink software. The WF comprises four DFIGs linked to a Point of Common Coupling (PCC), collectively delivering a capacity of 6 MW. These operations are evaluated across four distinct wind profiles. Figure 9 illustrates the progression of WS for each turbine, depicted as a scalar function evolving over time. The model is deterministically formulated as the sum of several harmonics, with an average speed of 8.2 m/s. A sampling time of 10e−4 s was adopted. The parameters used for GSC are: *ωs* = 2π50 rd/s, *Rg* = 2 mΩ, *Lg* = 5 mH. In addition, the parameters for WT are: Number of blades = 3, *R* = 35.25 m, *G* = 90, *J* = 1000 kg m^2^, *fv* = 0.0024 N m s^−1^, *V*_*d*_ = 4 m/s, *V*_*m*_ = 25 m/s. The parameters used for DFIG are: *P*_*n*_ = 1.5 MW, *v* = 12.5 m/s, *V*_*s*_ = 398/690 V, *I*_*n*_ = 1900 A, *f* = 50 Hz, *Ls* = 0.0137 H,* L*_*r*_ = 0.0136 H, *L*_*m*_ = 0.0135 H, *Rs* = 0.012 Ω, *Rr* = 0.021 Ω, p = 2, *J* = 1000 kg m^2^.

To rigorously assess our proposition, a comprehensive plan was formulated. This plan encompasses a series of meticulously crafted tests and scenarios that ensure the effectiveness of our research. Our approach amalgamates *Ps* and *Qs* management, alongside self-filtration management of WFs. This comprehensive strategy contributes to fulfilling the demands of the electrical grid while simultaneously safeguarding and enhancing energy quality, especially in scenarios involving non-linear load-induced disturbances.

### Implementing the management of *Ps* and *Qs* while ensuring low voltage ride through (LVRT)

A series of simulation scenarios has been meticulously conducted, divided into distinct periods. In each period, one of the three designated operating modes is engaged: MPPT, Delta, and Fault. The initiation of the fault command mode is automatic, triggered when a grid fault leads to a voltage drop.

The Danish grid code serves as the basis for the dip utilized in our study^83^. At 5 s into the simulation, a dip voltage of 20% transpires, followed by a more pronounced 70% voltage dip at the 7.5 s, as vividly depicted in Fig. 8.

In Fig. 8, it's evident that the grid current remains stable and consistent throughout, except during a fault occurrence, where it experiences a temporary decrease. Moving on to Fig. 11, we observe that the DC vector voltage of the primary unit closely tracks its specified reference value (*V*_*dc-ref*_ = 1200 V). However, within period 5, a minor peak is evident, measuring less than 10% of the estimated value. This particular elevation doesn't pose a significant risk to the DC amplitude. Notably, up until the 7.5 s, there's another peak exceeding 10% of the nominal value. This occurrence triggers the activation of the fault mode due to the potential threat it poses to the system.

**Figure 10:**
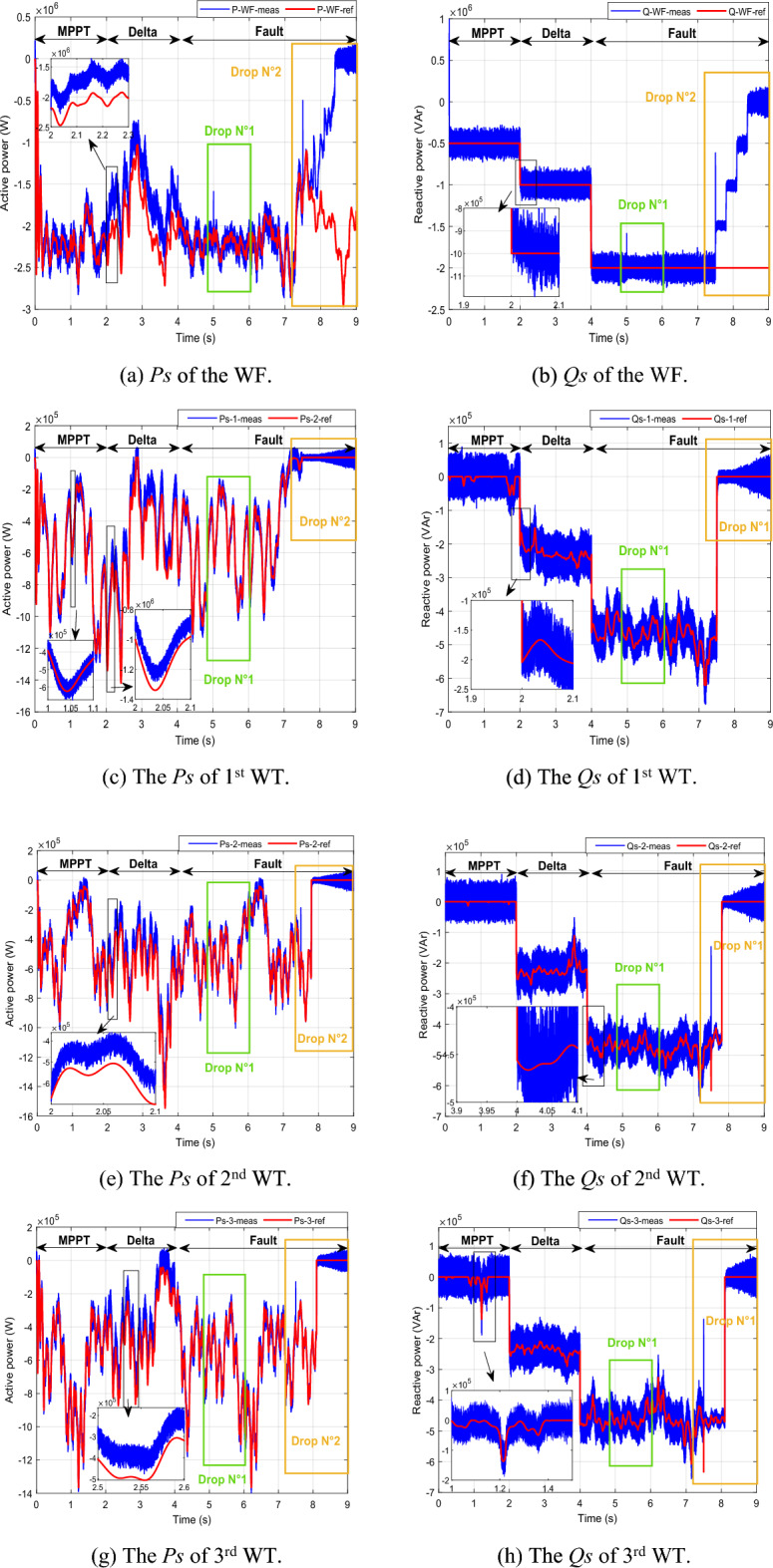

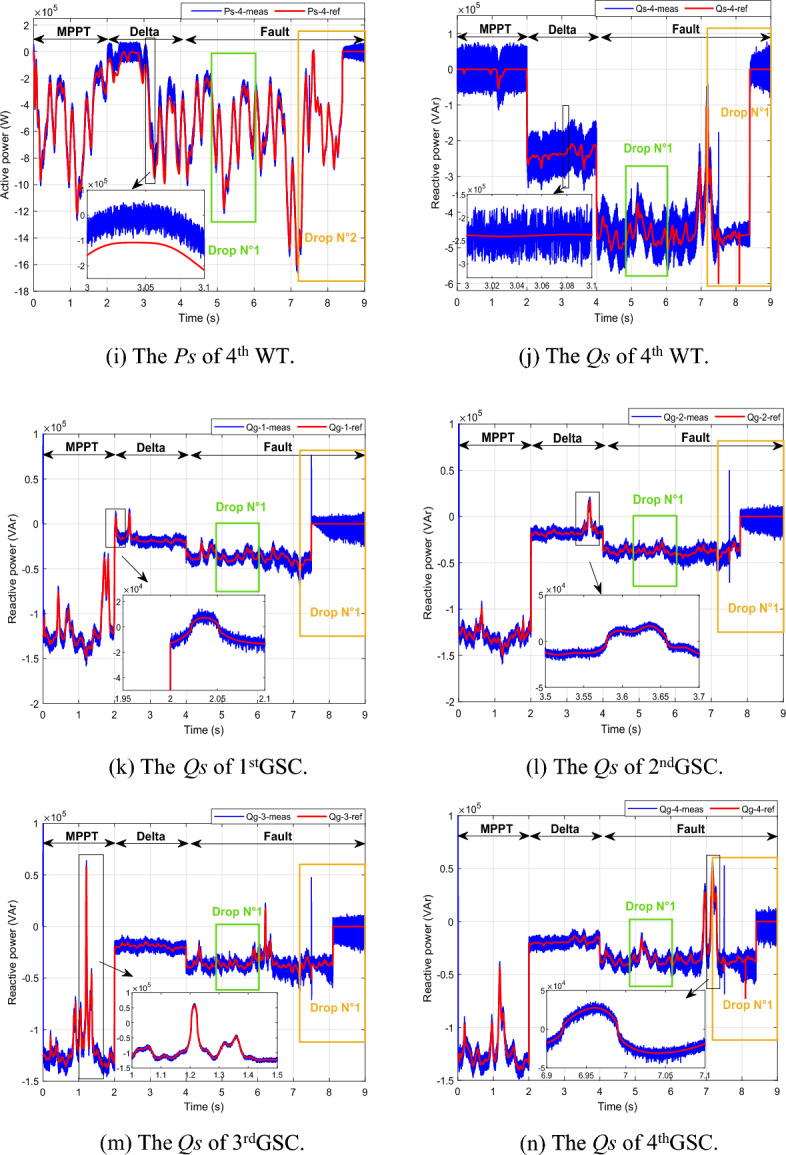
The simulation results for the scenario 1.

Presented in the following manner, this scenario unfolds:

- *Part 1* The MPPT command mode is engaged over a span of 2 s (from 0 to 2 s). As depicted in Fig. 10, the *Ps* produced at the PCC is the accumulation of the optimal *Ps* originating from the four generators showcased in Fig. 10a, c, e, i. In this period, the *Qs* is set at − 0.5 MVAR. Remarkably, despite variations in WS (Fig. 9), both the *Ps* and *Qs* at the PCC impeccably adhere to their reference values, as visualized in Fig. 10a, b. Each generator contributes proportionally based on its available power capacity. Notably, Fig. 10e reveal that the second WT is unable to fulfill the prescribed *Ps* demand. Nevertheless, this shortfall in power is effectively compensated for by the output of the first, third, and fourth generators.
- *Part 2* The "Delta" mode is initiated for a duration of 2 s (from 2 to 4 s). In this phase, the WF generates the necessary *Ps*, deliberately operating below its maximum power capacity. Simultaneously, the capability to generate *Qs* is also harnessed. This specific period serves to mitigate issues arising from the WF experiencing elevated WSs, which can cause frequency and voltage deviations within the electrical grid.
- *Part 3* The fault control mode is seamlessly triggered after a 5-s interval, initiated by the imposition of a voltage dip of 20% that endures for less than 1 s. Subsequently, this is followed by the application of a more pronounced 70% voltage drop at 7.5 s. This automatic activation of the fault operating mode ensures a swift response to adverse conditions within the system (Fig. 8). When encountering a 20% voltage drop within a 1 s interval, the effectiveness of the designed technique becomes evident in successfully averting this peak. This proficiency remains consistent even as the voltage drop is further intensified, ultimately reaching a level of 70%. Under these circumstances, the WTs necessitate disconnection through the automatic system protection. This particular scenario culminates with the sequential disconnection of the four generators from the grid at 7.5 s, 7.8 s, 8.1 s, and 8.4 s, as visually represented in Fig. 10c–n. As the scenario unfolds, the *Ps* and *Qs* observed at the PCC, depicted in Fig. 10a, b undergo a gradual reduction, ultimately tapering to zero.

The configuration of the central supervisory unit within the farm serves the purpose of fulfilling the comprehensive production power plan stipulated by the network manager. This unit also facilitates the transmission of crucial information to the local supervision units, enabling precise control over each individual WT.

The outcomes of the simulation incontrovertibly showcase that the synergy between the supervisory system, the proportional distribution algorithm, and the power control executed at the PCC collectively form a robust framework, effectively ensuring the stable and uninterrupted WF operation. Within this framework, the FS-MPC controller notably ensures exceptional levels of traceability, performance, reliability, and robustness.

### Performance comparison under symmetrical and asymmetrical fault

**Figure 11:**
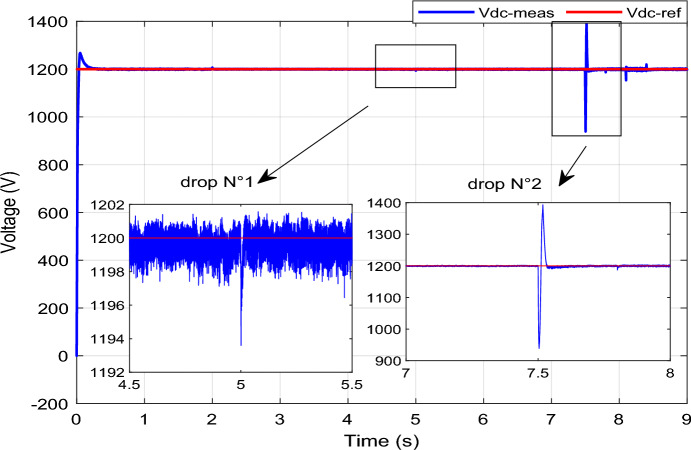
DC bus voltage of 1st WT.

To assess the efficacy of the control strategy in enhancing performance and ensuring uninterrupted fulfilment of requirements within the electrical grid, a comprehensive analysis is conducted by simulating both symmetric and asymmetric grid fault scenarios on a DFIG. Throughout this study, the wind turbine's estimated speed is set at 12.5 m/s, with the reference *Ps* and *Qs* undergoing unit steps (Fig. 11).

**Figure 12:**
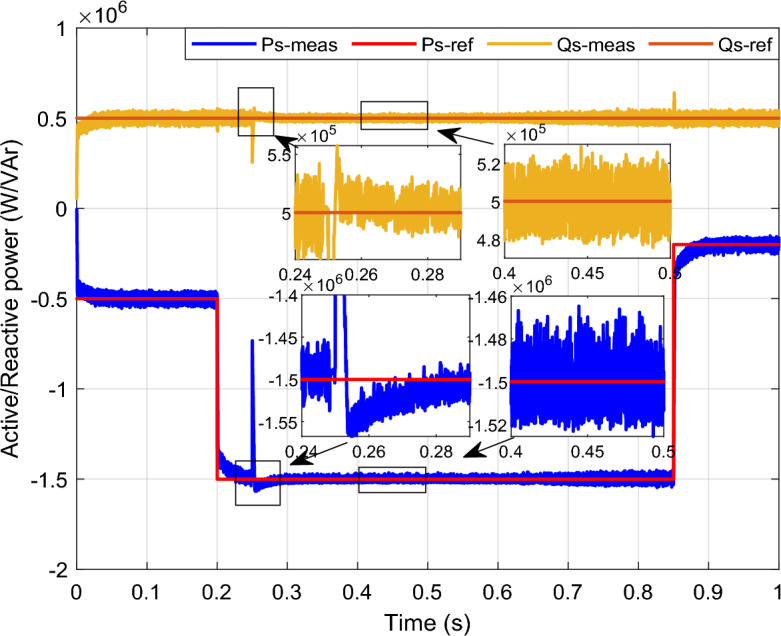
*Ps* and *Qs* with symmetrical (L–L–L) fault.

**Figure 13:**
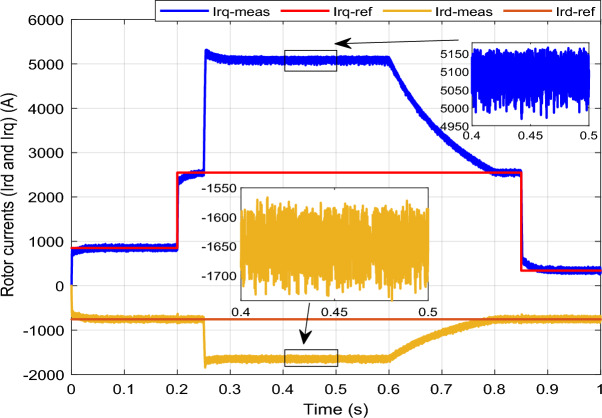
*I*_*rq*_ and *I*_*rd*_ with symmetrical (L–L–L) fault.

**Figure 14:**
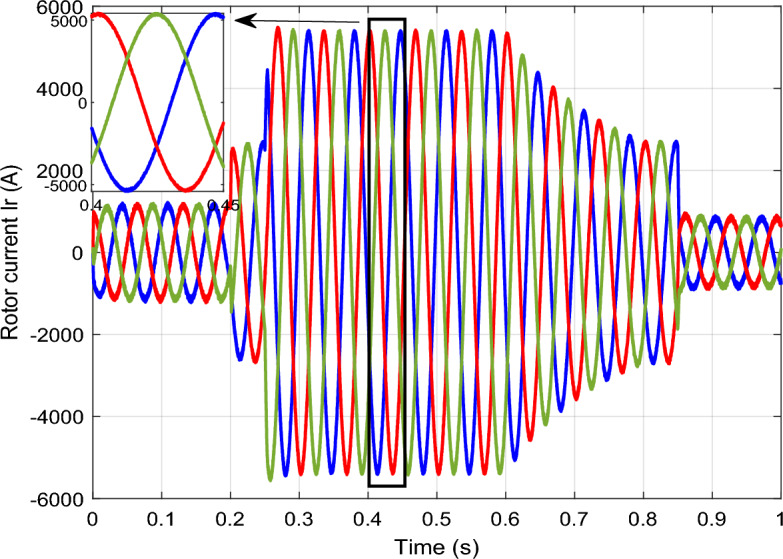
*I*_*r*_ with symmetrical (L–L–L) fault.

**Figure 15:**
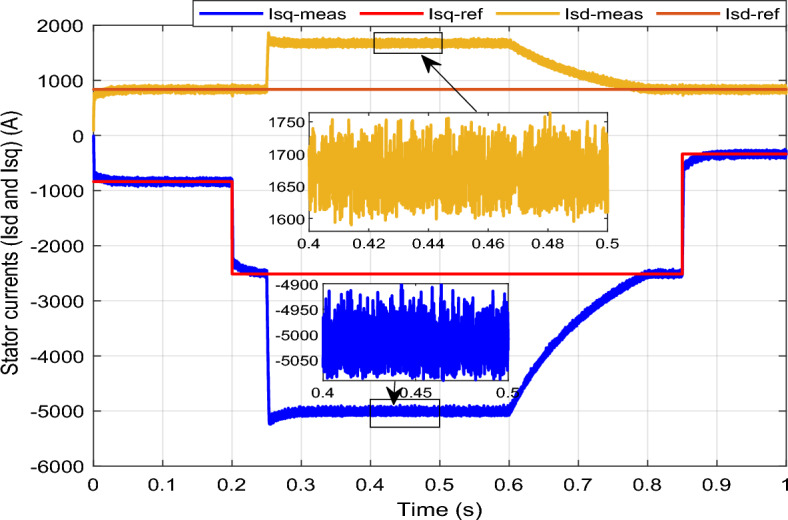
*I*_*sq*_ and *I*_*sd*_ with symmetrical (L–L–L) fault.

**Figure 16:**
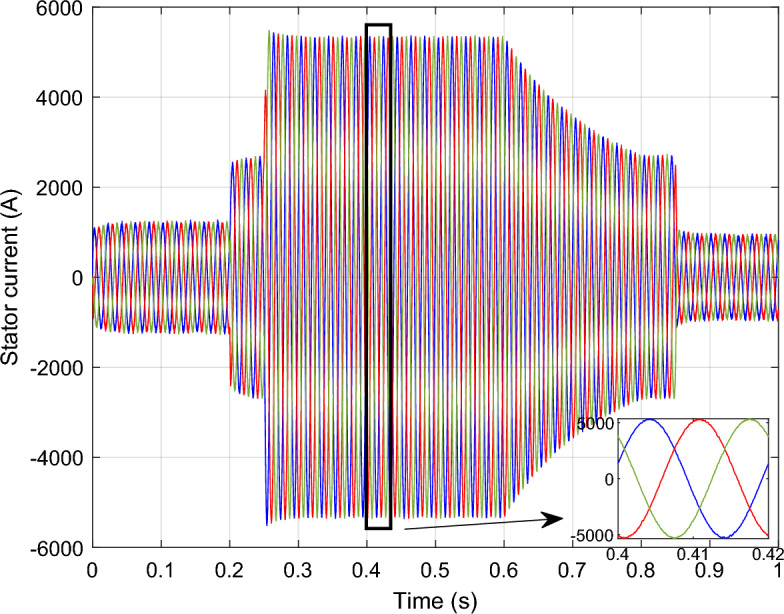
*I*_*s*_ with symmetrical (L–L–L) fault.

- symmetrical three-phase (L–L–L) fault:

In this case study, a symmetrical (L–L–L) fault scenario is considered to verify the efficacy of the control strategy of the wind system. An L–L–L fault (drop = 70%) occurs at t = 0.25 s on three phases, and the duration of the fault is 0.8 s. Figures 12, 13, 14, 15 and 16 shows the *Ps* and *Qs* and instantaneous rotor and stator current waveforms for the control method proposed.

**Figure 17:**
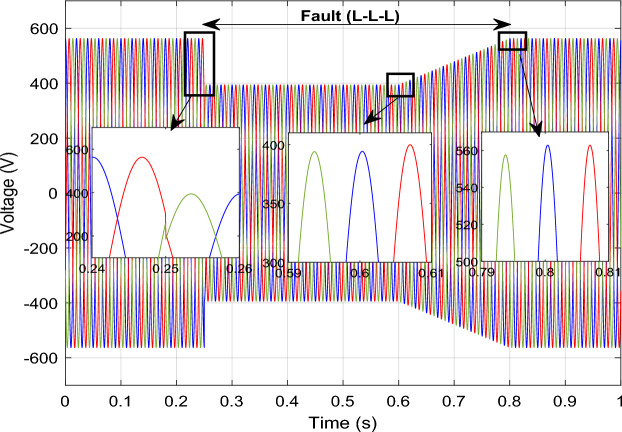
Voltage in symmetrical (L–L–L) fault.

The outcomes of the validity test are visually represented in Figs. 12, 13, 14, 15, and 16. Notably, the overshoots in power exhibited by the proposed controller in response to the decline are deemed largely acceptable (Fig. 12), especially when contrasted with the substantial drop ratio. It is noteworthy, however, that the current waveforms display a notable improvement compared to the outcomes in reference^84^. In the referenced study, the proposed control exhibited a 100% non-waveform response, whereas the proposed method in this study demonstrates a significant enhancement in LVRT performance by effectively mitigating overcurrent induced by grid disturbances. Figure 17 represents the voltage profile in the case of a symmetrical (L–L–L) fault, where it is noted that the voltage amplitude changes in the case of a (L–L–L) fault. The voltage profile remains sinusoidal but unbalanced. The largest voltage amplitude is 559 V and the minimum is 400 V.

**Figure 18:**
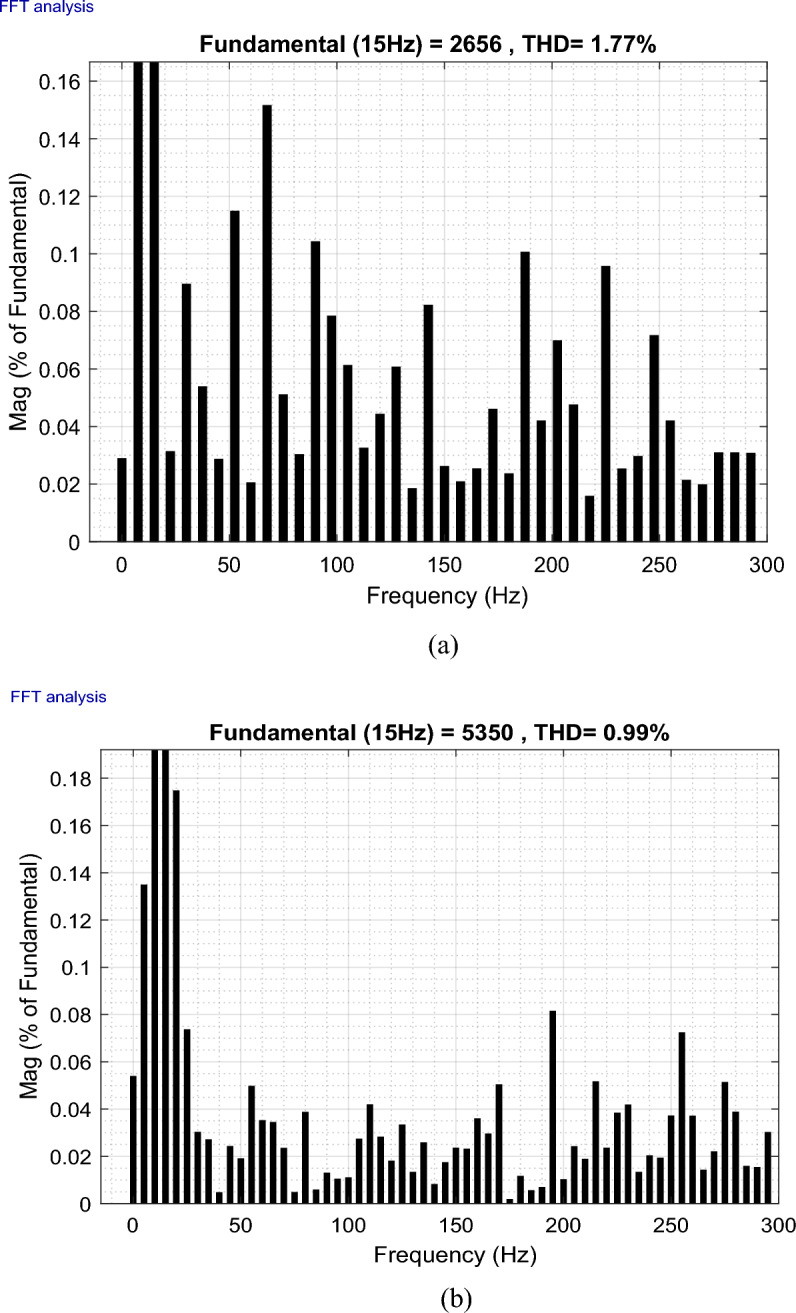
THD values of the *I*_*r*_ [(**a**) before fault and (**b**) After fault].

**Figure 19:**
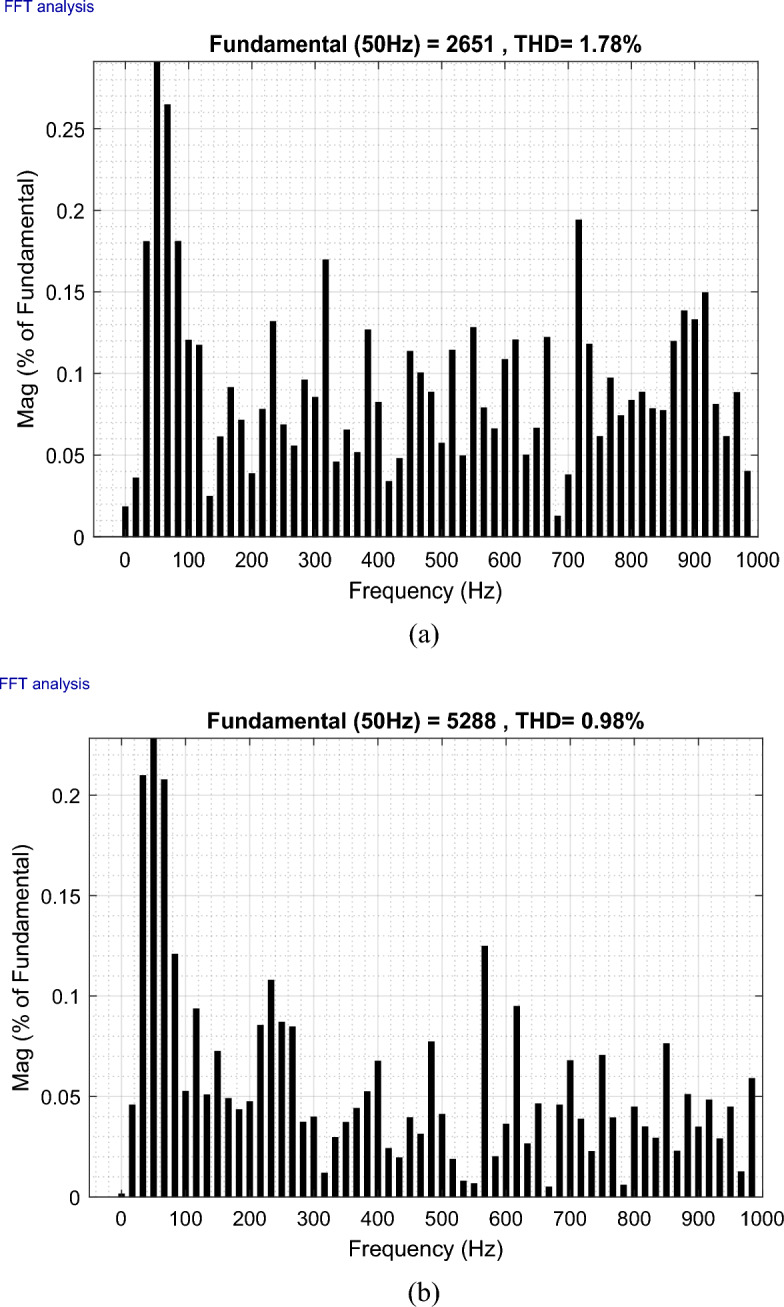
THD values of the *I*_*s*_ [(**a**) before fault and (**b**) After fault].

The THD value of the rotor and stator current of the DFIG is represented in Figs. 18 and 19 (before and after fault), and the numerical values for each current (*I*_*r*_ and *I*_*s*_) are extracted in Table 1. The THD value of the current remains the same as the values after the fault occurs, which indicates the effectiveness of the proposed strategy in dealing with the fault. The THD for the currents are 1.77% and 1.87% for *I*_*r*_ and *I*_*s*_, respectively. On the other hand, Table 2 represents the basic signal amplitude value for both rotor and stator current. It is noted from this table that the signal amplitude increased significantly after the fault for *Ir* and *Is*, by a percentage estimated at 50%. This increase is due to the symmetrical fault, but despite the increase, the proposed control maintains energy quality.

**Table 1:** Ratios and values THD of current.

	THD (%)		Ratios (%)
	Before fault	After fault	
*Ir*	1.77	0.99	44
*Is*	1.87	0.98	48

**Table 2:** Signal amplitude values fundamental of current.

	Amplitudes (A)		Ratios (%)
	Before fault	After fault	
*Ir*	2656	5350	50
*Is*	2651	5288	50

Table 3 provides the ripple values for *Ps* and *Qs* both before and after the fault, along with the calculated reduction ratios for *Ps* and *Qs* ripples. A noteworthy observation from the table is that the power ripples are diminished after the fault in comparison to the pre-fault conditions. This outcome serves as evidence of the efficacy of the proposed strategy in reducing the amplitude of *Ps* and *Qs* ripples, as corroborated by the calculated reduction ratios. Specifically, the reduction percentage for *Ps* stands at 67%, while the ripple reduction ratio for *Qs* is calculated at 51%. These results underscore the effectiveness of the proposed strategy in mitigating power fluctuations, enhancing the stability and reliability of the system.

b. asymmetrical fault:

**Table 3:** Ratios and values of ripples for DFIG power.

	Ripples		Ratios (%)
	Before fault	After fault	
*Ps*	90,000	30,000	67
*Qs*	91,000	45,000	51

In this particular case study, the asymmetrical fault is deliberately categorized into two distinct scenarios:

1. A single-phase fault from phase A to ground (L–G) is intentionally introduced at the PCC at time (t) = 0.1 s, and it continues for a duration of 0.45 s.
2. A two-phase fault involving phases B and C to ground (L–L–G) is intentionally induced at the PCC at time (t) = 0.55 s, with a duration extending over 0.9 s.

These intentional fault scenarios are designed to assess and analyze the system's response under asymmetrical conditions, providing valuable insights into the robustness and performance of the control strategy.

**Figure 20:**
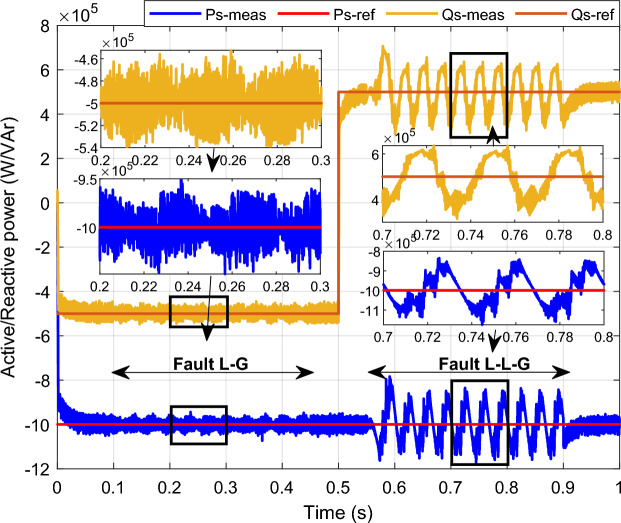
Ps and *Qs* with asymmetrical fault.

**Figure 21:**
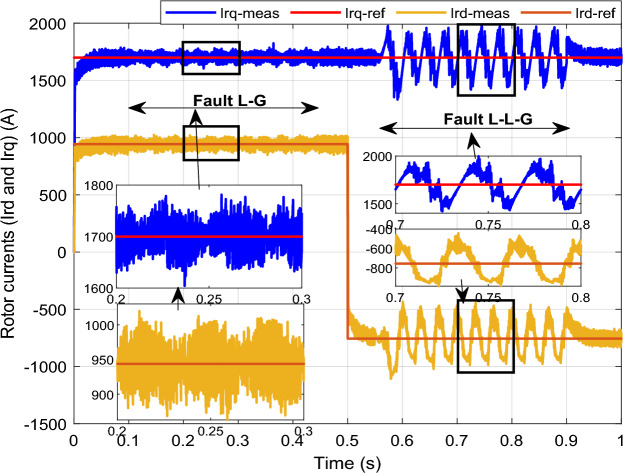
*I*_*rq*_ and *I*_*rd*_ with asymmetrical fault.

**Figure 22:**
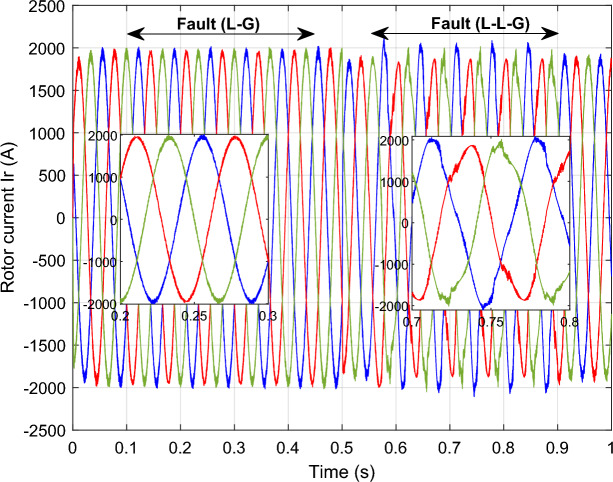
*I*_*r*_ with asymmetrical fault.

**Figure 23:**
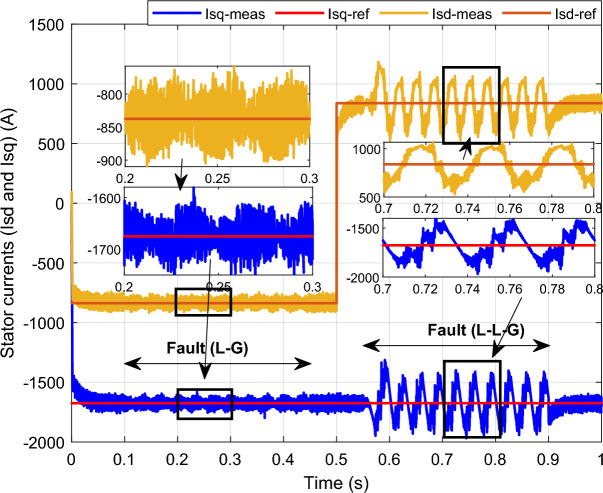
*I*_*sq*_ and *I*_*sd*_ with asymmetrical fault.

**Figure 24:**
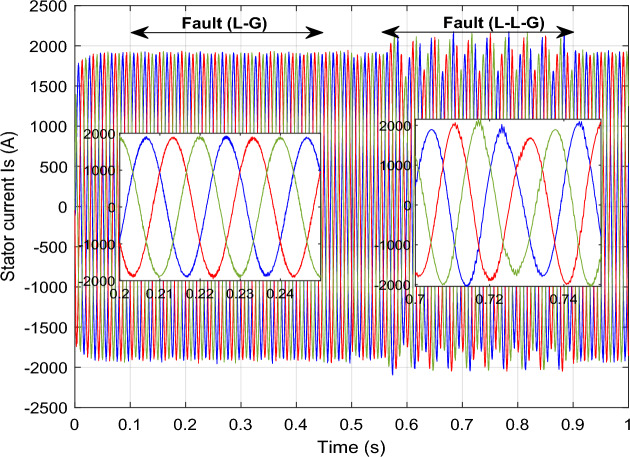
*I*_*s*_ with asymmetrical fault.

**Figure 25:**
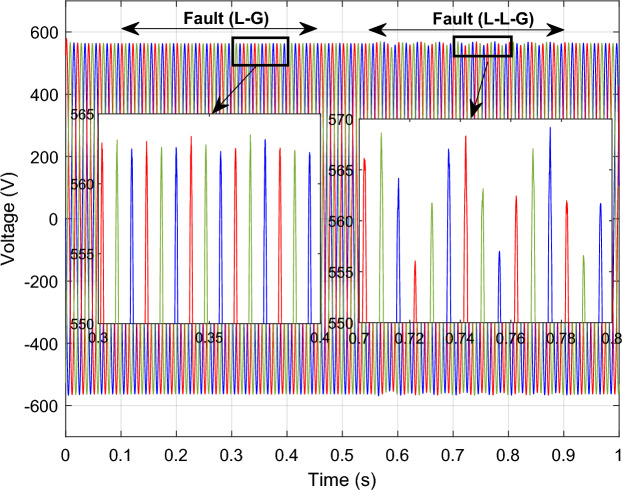
Voltage in asymmetrical fault.

The responses and dynamic behaviors of the instantaneous *Ps*, *Qs*, rotor current, stator current, and voltage are depicted in Figs. 20, 21, 22, 23, 24 and 25, respectively.

The obtained results reveal that the fault (L-G), characterized by a single-phase fault from phase A to ground, does not exhibit any discernible impact on the power ripples (*Ps* and *Qs*) (Fig. 20) or the rotor and stator currents (*Ir*, *Irq*, *Ird*, *Is*, *Isq*, and *Isd*) (Figs. 21, 22, 23, 24). This observation stands in stark contrast to the outcomes reported in reference^84^, where the results indicated a substantial impact on the waveforms of currents under similar fault conditions. The disparity in results underscores the effectiveness of the proposed control strategy in mitigating the impact of the fault (L–G) and maintaining the stability of power ripples and current waveforms. This suggests an improvement in system performance and fault tolerance compared to the reference^84^, highlighting the efficacy of the implemented control mechanisms.

When the fault (L–L–G) occurs, the graphical representations illustrate that the transient response and behavior of both *Ps* and *Qs* (Fig. 20) exhibit ripples, indicative of the pronounced influence of the fault. However, despite the impact of the fault, the rotor (Figs. 21, 22) and stator current (Figs. 23, 24) exhibit smoother and less oscillatory patterns in comparison to the findings in reference^24^. Noteworthy is the observation that, despite faults occurring in two phases, the control mechanism maintains convergence and does not compromise its stability. Figure 25 represents the voltage profile in the case of an asymmetrical fault. It is noted that the voltage amplitude is greatly affected in the case of the fault (L–L–G) compared to the fault (L–G) while the voltage profile remains sinusoidal. In the case of fault (L–G), the largest voltage value was 563 V and 568 V in the case of fault (L–L–G). So the voltage amplitude increased in the case of fault (L–L–G).

**Figure 26:**
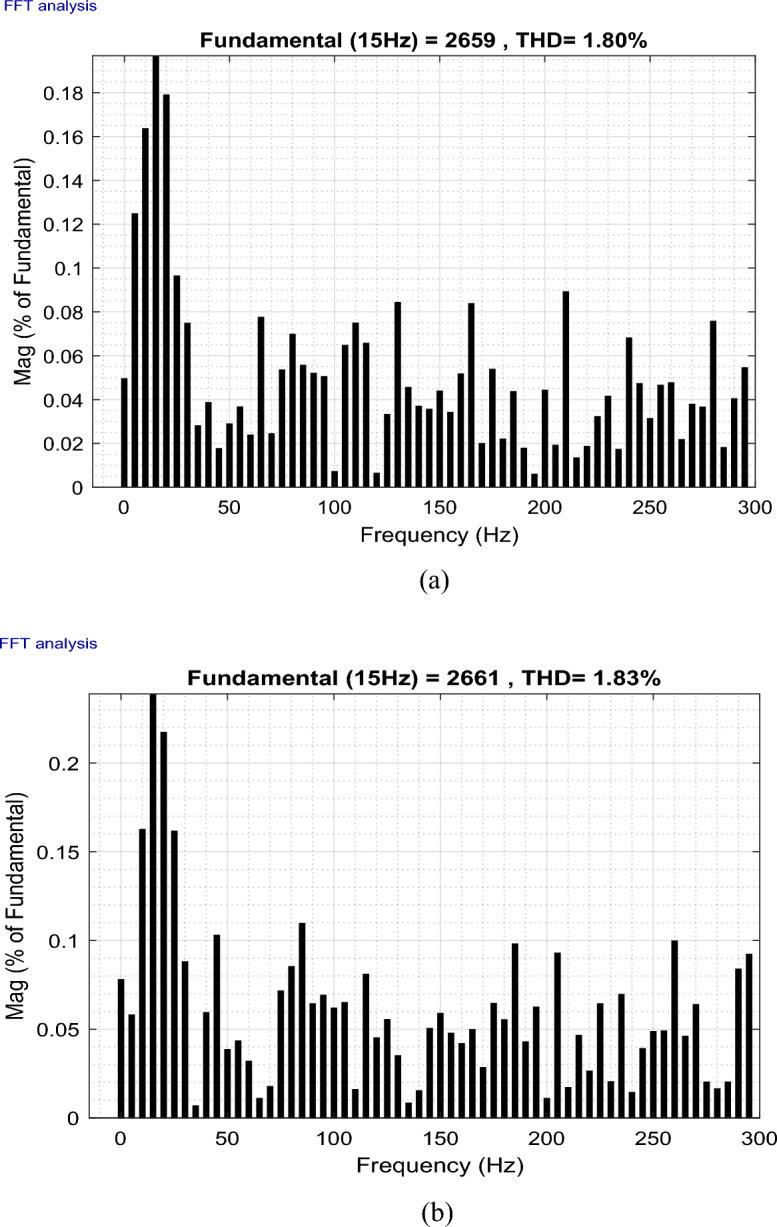
THD values of the *I*_*r*_ [(**a**, **b**) before fault at 0.2 s, and 0.65 s, respectively].

**Figure 27:**
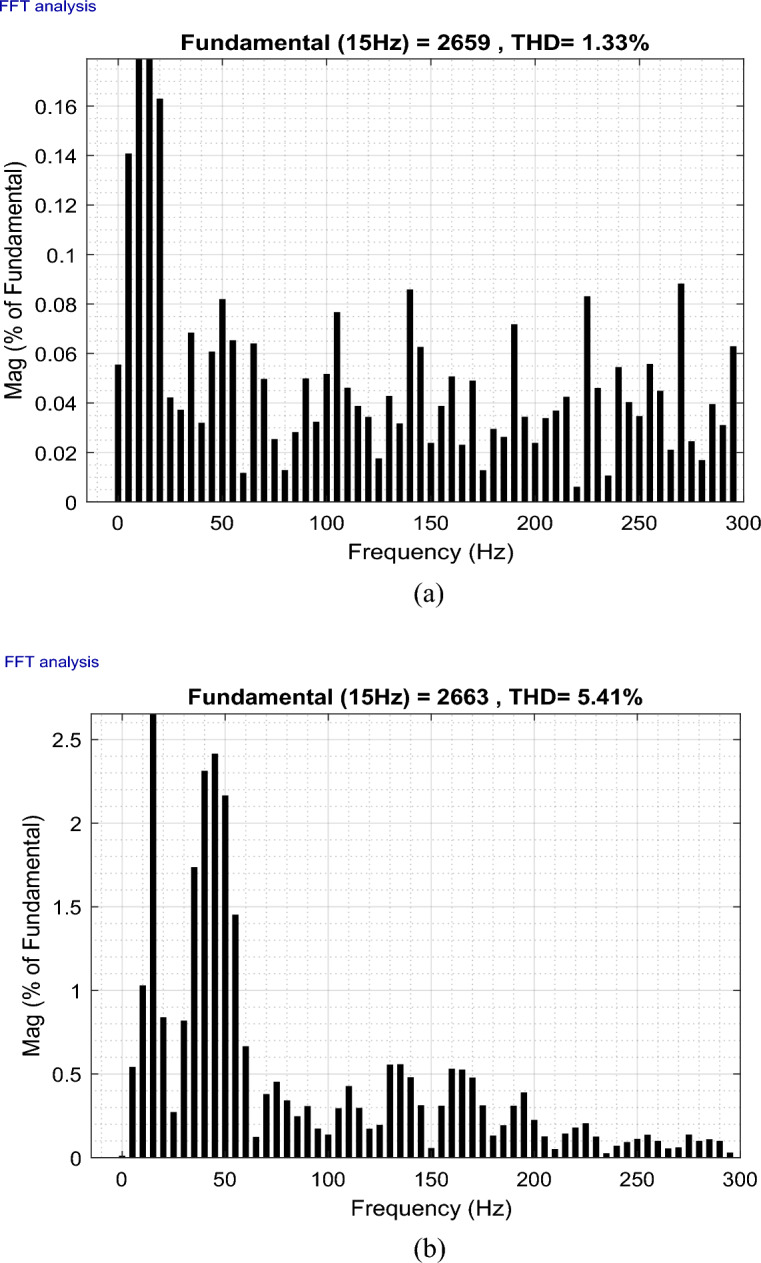
THD values of the *I*_*r*_ [(**a**, **b**) after fault at 0.2 s, and 0.65 s, respectively].

**Figure 28:**
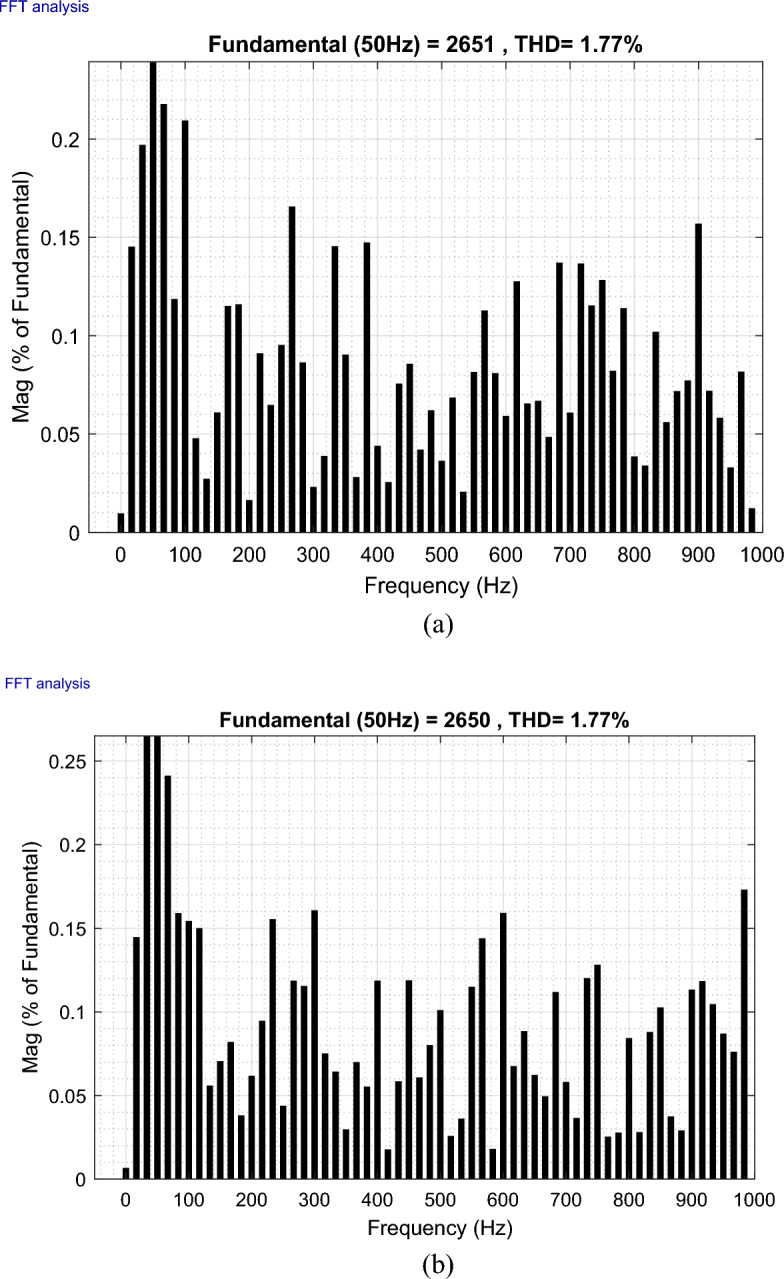
THD values of the *I*_*s*_ [(**a**, **b**) before fault at 0.2 s, and 0.65 s, respectively].

**Figure 29:**
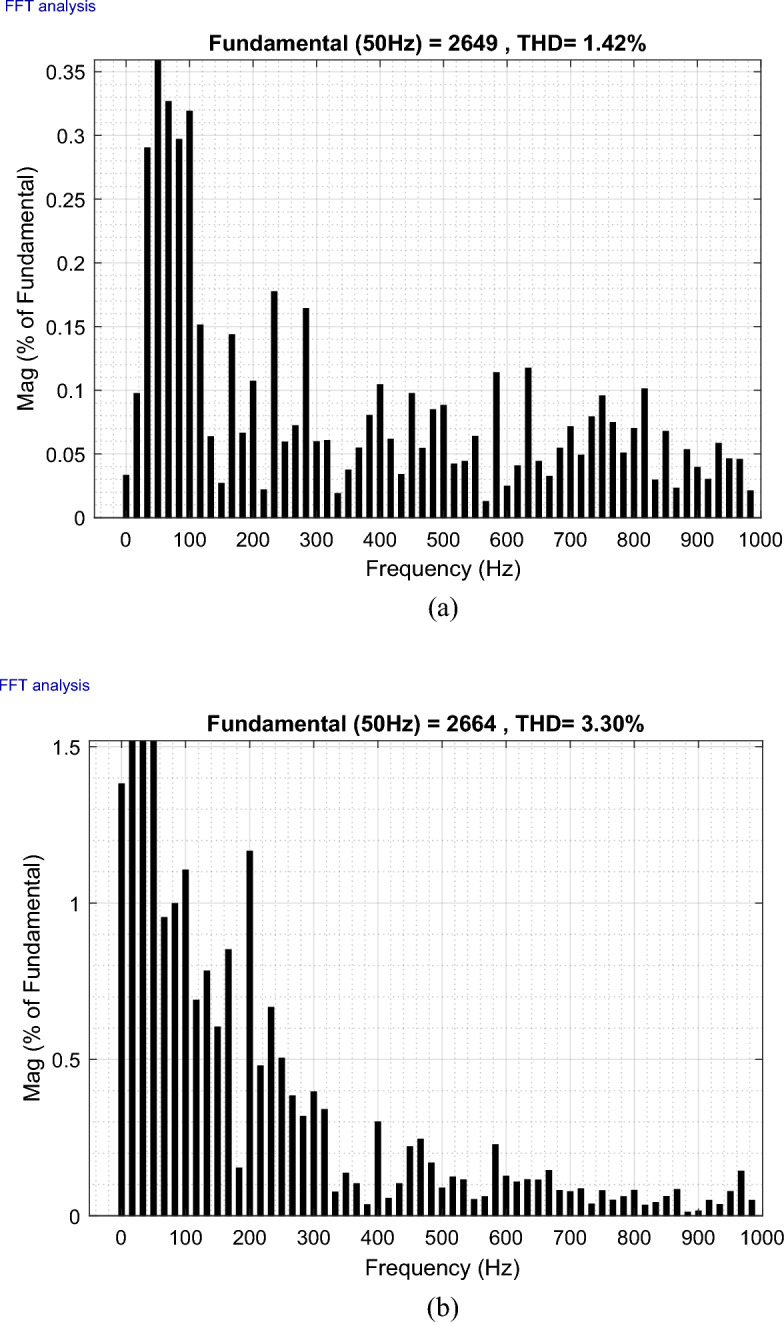
THD values of the *I*_*s*_ [(**a**, **b**) after fault at 0.2 s, and 0.65 s, respectively].

Figures 26, 27, 28 and 29 depict the THD for currents *I*_*r*_ and *I*_*s*_ both before and after the fault occurrence. Notably, following the occurrence of the initial fault (L-G), the THD values exhibit remarkable stability, with a percentage change estimated at 26% for *I*_*r*_ and 20% for *I*_*s*_, as outlined in Tables 4 and 5. It is noteworthy that despite the fault, the numerical value of THD remains relatively constant. Furthermore, in this particular scenario, the ripples observed in the *Ps* and *Qs* experienced a decline, showcasing percentage changes of 22% and 19%, respectively, as indicated in Table 8. This observation adds depth to our understanding of the system's response to the fault, emphasizing the resilience and stability of the studied parameters.

**Table 4:** Ratios and values THD of rotor current .

Time (s)	THD (%)		Ratios (%)
	Before fault	After fault	
0.2	1.80	1.33	26
0.65	1.83	5.41	66

**Table 5:** Ratios and values THD of stator current.

Time (s)	THD (%)		Ratios (%)
	Before fault	After fault	
0.2	1.77	1.42	20
0.65	1.77	3.3	46

The emergence of the second fault (L–L–G) introduces a discernible ripples in both *Ps* and *Qs*, marking a substantial change estimated at 79% for each, as detailed in Table 8. Despite the pronounced ripple in power, it is noteworthy that the power quality remains within acceptable limits for both currents *I*_*r*_ and *I*_*s*_. The respective changes are estimated at 66% for *I*_*r*_ and 46% for *I*_*s*_ when compared to the power quality levels observed before the fault occurred (Tables 4, 5).

Intriguingly, despite the dynamic alterations in power parameters, the fundamental signal amplitude values of *I*_*r*_ and *I*_*s*_, as outlined in Tables 6 and 7, remain unaltered in both scenarios. This implies that, despite the significant fluctuations in *Ps* and *Qs*, the core characteristics of the current waveforms in terms of amplitude persist, underscoring a certain degree of robustness and stability in the system under examination (Table 8).

**Table 6:** Signal amplitude values fundamental of rotor current.

Time (s)	Amplitudes (A)		Ratios (%)
	Before fault	After fault	
0.2	2659	2659	0
0.65	2661	2663	0

**Table 7:** Signal amplitude values fundamental of rotor current.

Time (s)	Amplitudes (A)		Ratios (%)
	Before fault	After fault	
0.2	2650	2649	0
0.65	2650	2664	1

**Table 8:** Ratios and values of ripples for DFIG power.

	Ripples			Ratios (%)	
	Before fault	After fault		L–G	L–L–G
		L–G	L–L–G		
Ps	90,000	70,000	440,000	22	79
Qs	91,000	76,600	441,800	19	79

### Self-filtering of WF

This scenario delves into the concept of self-filtering within a WF, focusing on testing the efficacy of the proposed supervisory unit when employed to filter the grid electrical in scenarios involving non-linear loads with substantial currents. To ensure the precision of our findings, the *Ps* and *Qs* references were adopted as unit step, all the while maintaining a consistent WS of 12.5 m/s. This setup guarantees the reliability and accuracy of our results in assessing the performance of the proposed supervisory unit.

**Figure 30:**
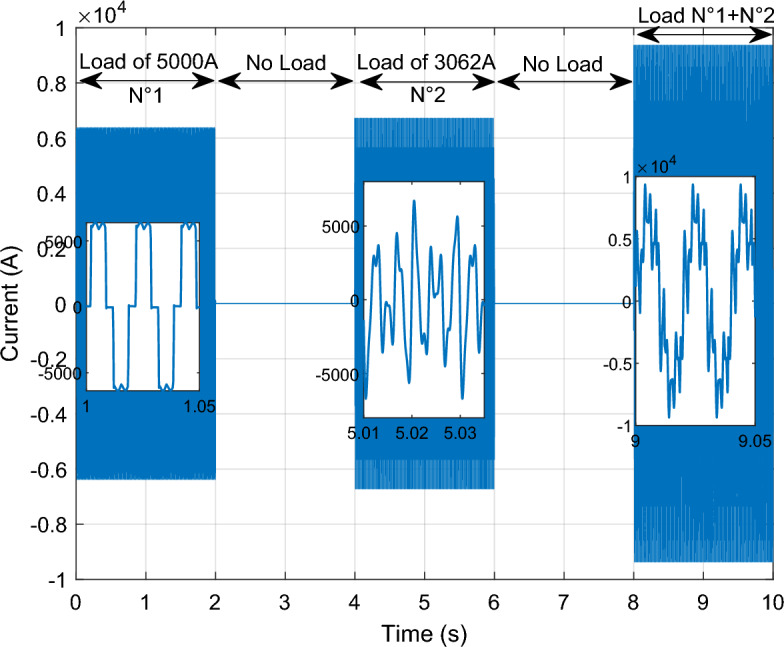
Current absorbed by a non-linear load.

Figure 30 illustrates the nonlinear load employed in our study. Initially, a load in the form of a rectifier is connected at 0 s, with an effective value of 5000 A. At the 2 s, no harmonics are injected into the electrical grid. Subsequently, at 4 s, a distinct load is introduced, differing from the initial one. This load arises from the injection of odd harmonics, specifically the 5th, 7th, 11th, and 13th harmonics, and consumes a current of 3062 A. From 4 to 6 s, the load becomes nil, indicating the absence of non-linear loads. Upon reaching the 8 s, the third load is connected, resulting from the combination of the first load (5000 A) with the second load (3062 A).

Given the substantial impact of these loads, it's evident that they carry significant impact and cannot be adequately addressed by a single DFIG unit alone; the WF involvement is essential. With this consideration, we imposed a maximum filtering capacity of 1350 A for each DFIG unit.

**Figure 31:**
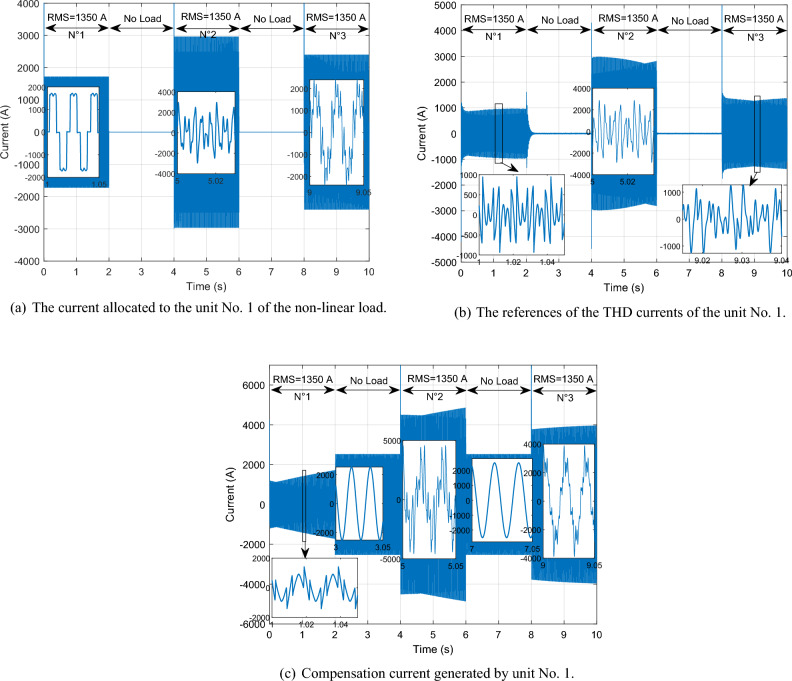
Simulation results of the unit No. 1 with and without active filtering function.

**Figure 32:**
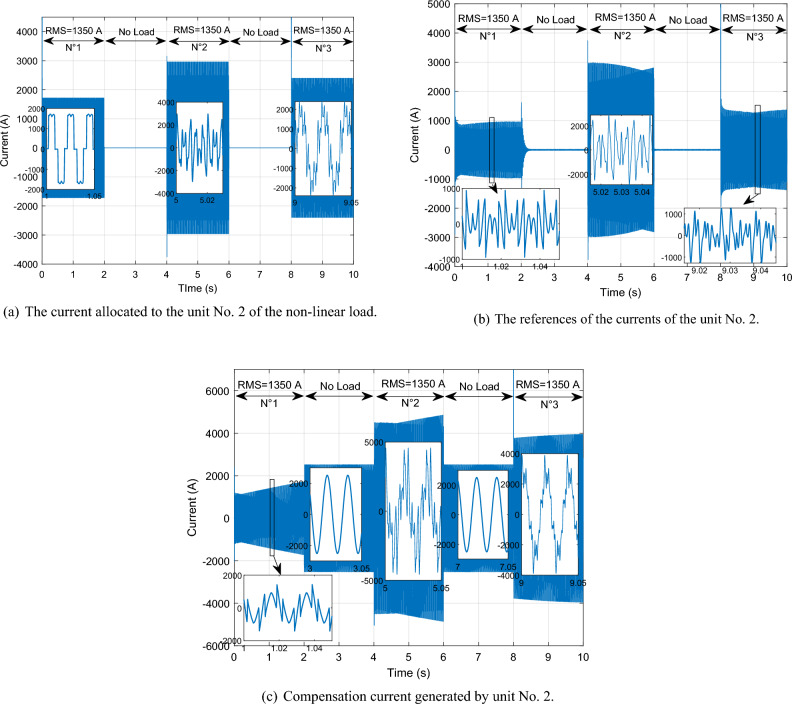
Simulation results of the unit No. 2 with and without active filtering function.

**Figure 33:**
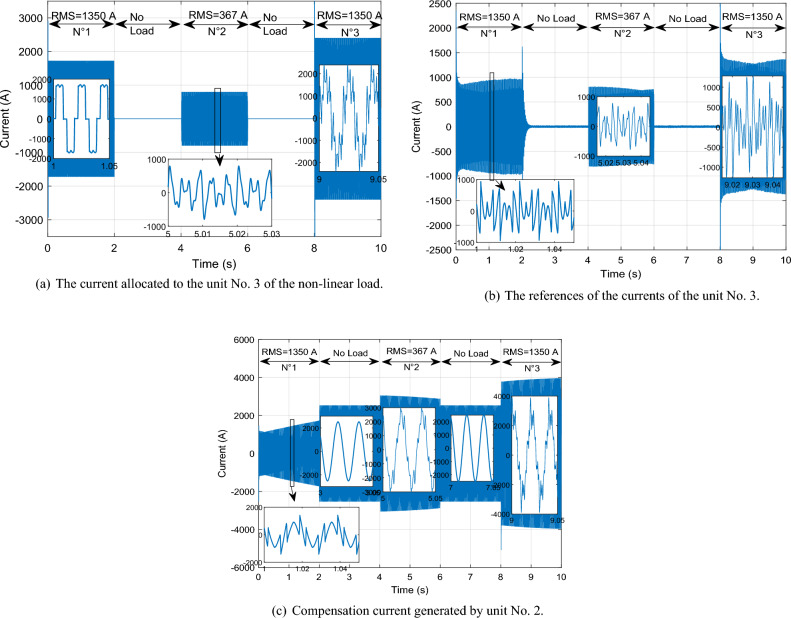
Simulation results of the unit No. 3 with and without active filtering function.

**Figure 34:**
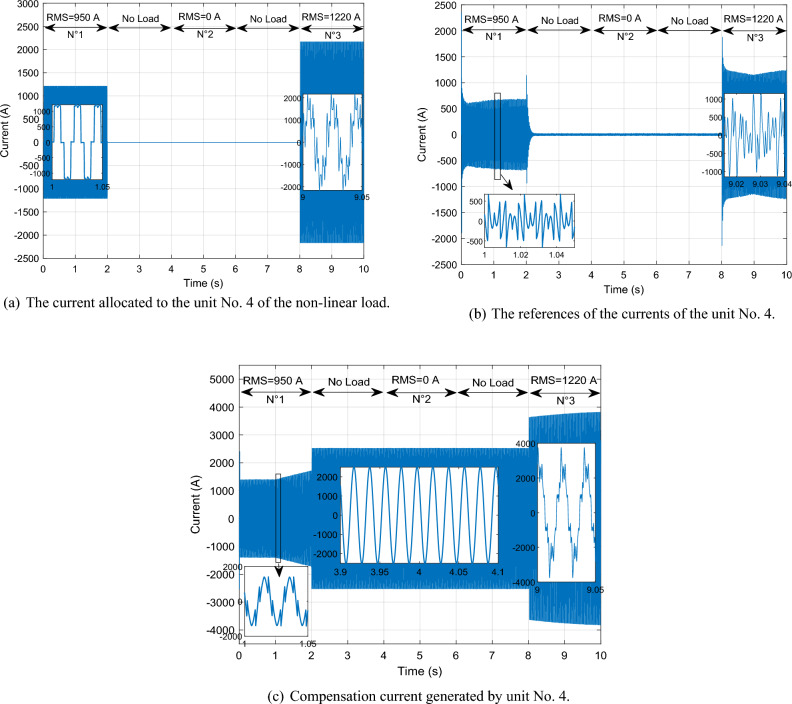
Simulation results of the unit No. 4 with and without active filtering function.

**Figure 35:**
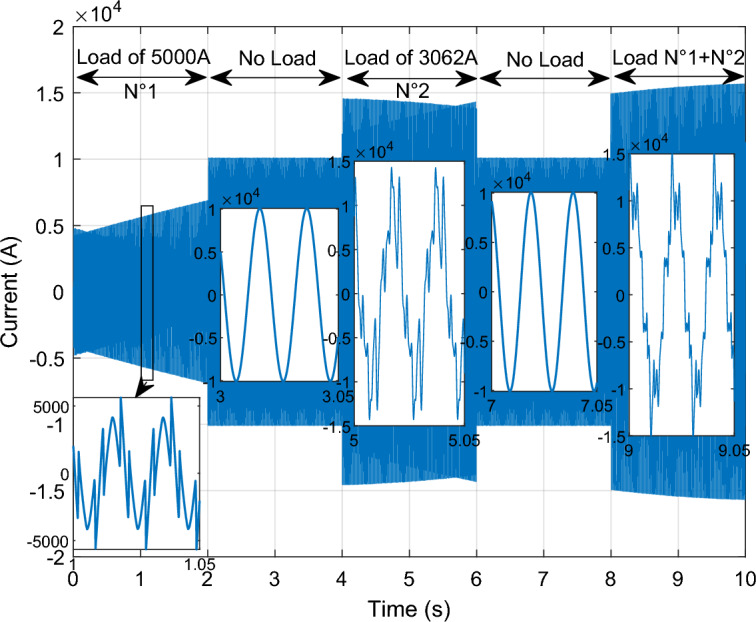
Shape of WF currents.

**Figure 36:**
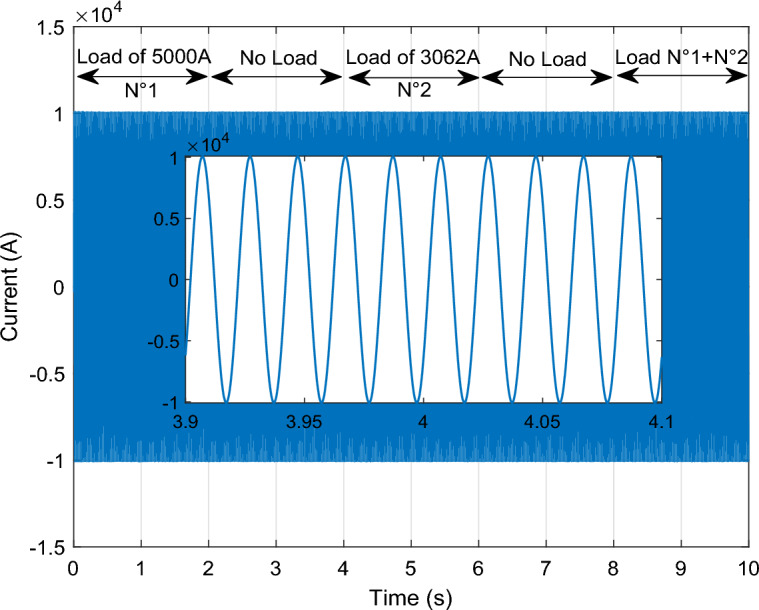
Shape of grid current after filtering.

By implementing this approach, the excess current from the first DFIG is effectively filtered by the second unit. Subsequently, the residual surplus from the second DFIG is further filtered by the third, and this cascade of filtering continues to the fourth unit. This strategic principle allows for the collaborative contribution of all four units within the process. Through this systematic and sequential procedure, the results shown in Figs. 31, 32, 33, 34, 35 and 36 were obtained.

Based on the findings presented in Figs. 31, 32, 33, 34, 35 and 36, the time interval can be categorized into four distinct segments:

- [from 0 to 2 s]: the proposed supervisory unit performs an assessment of the electrical grid. At this interval, the effective value of the load current is measured to be 5000 A. Subsequently, the supervisory unit designates responsibilities to four units within the WF for the purpose of electrical grid filtering.

The first, second, and third units are directed to operate at a capacity of 1350 A each. The remainder of the 5000 A is 950 A allocated to the fourth unit. Throughout this duration, a THD test was conducted, yielding a higher result of 56.87%. However, following the implementation of the grid filtering strategy, this THD value significantly dropped to a mere 0.32%. This remarkable reduction underscores the effectiveness of the proposed intervention and its positive impact on the overall grid quality.

- [from 2 to 4 s] and [from 6 to 8 s]: Within these intervals, the electrical grid is in a stable state, during which the supervisory unit functions exclusively to control the *Ps* and *Qs* of the WF.
- During the time interval from 4 to 6 s, an examination of the current load (RMS = 3062 A) was conducted. It was decided to delegate this task to three units on the farm to carry out the grid filtering process. The first and second units each handled 44% of the load current, while the remaining 12% was managed by the third unit. As for the fourth unit, it was not included in the operation. Notably, during this period, the THD test produced a relatively high result of 43.05%. However, after the filtering process was implemented, this value decreased significantly to 0.29%, indicating the effectiveness of the designed solution.
- Between 8 and 10 s, a specific scenario unfolded. The first and second loads were consolidated to create a load with a value of 5270 A. To manage this load, a supervision unit employs four units from the farm. The first, second, and third units each worked on 26% of the load, with the remaining 22% allocated by the supervision unit to filter unit No. 4. Interestingly, the inclusion of the third load resulted in a decline in power quality, with a THD equal to 27.50%. However, this figure was subsequently reduced to a remarkable 0.26% thanks to the implementation of the designed algorithm.

**Figure 37:**
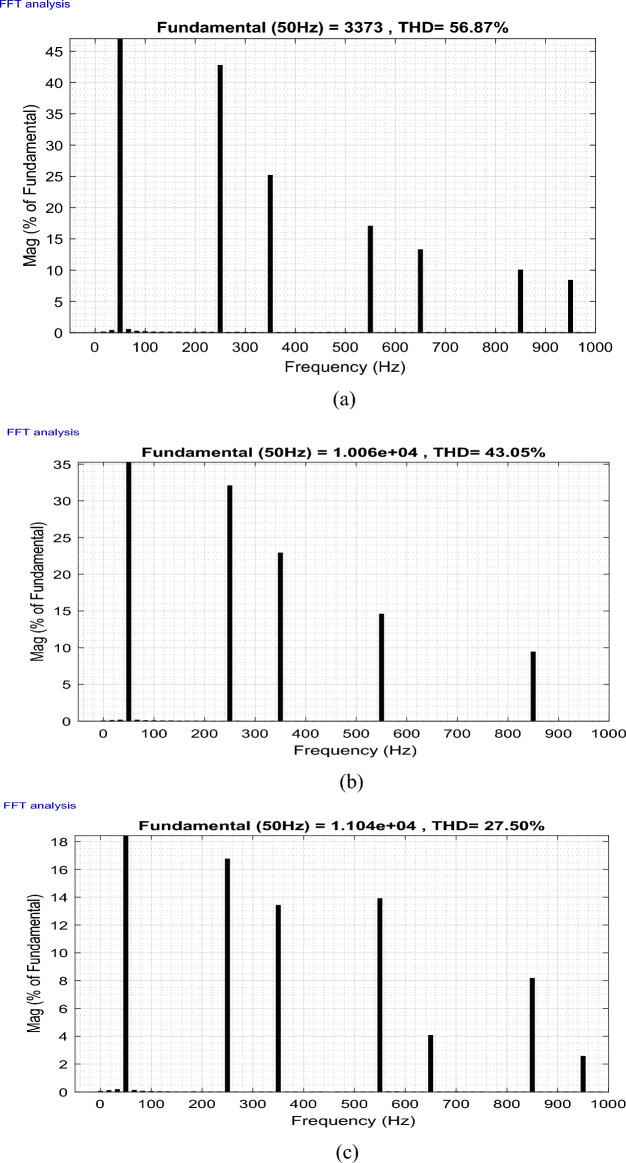
Spectrum of grid current before harmonic current compensation: (**a**) at 1 s; (**b**) at 5 s; (**c**) at 9 s.

**Figure 38:**
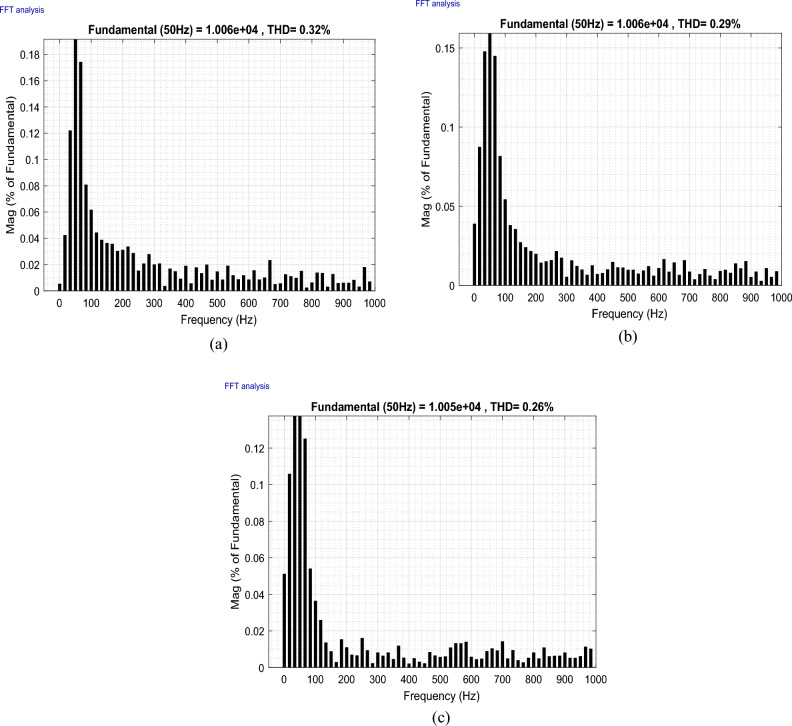
Spectrum of grid current after harmonic current compensation: [(**a**) at 1 s; (**b**) at 5 s; (**c**) at 9 s].

The THD value of the current is represented in Figs. 37 and 38 (before and after the harmonic current compensation), and the numerical values at different time points (1 s, 5 s and 9 s) are extracted in Table 9. The THD value of current is much lower after applying the filtering process (the proposed strategy), which indicates the effectiveness of the proposed strategy in improving the quality of the current. The THD of current reduction ratios were 99.43%, 99.32%, and 99.05% at 1 s, 5 s, and 9 s, respectively. On the other hand, Table 10 represents the amplitude value of the fundamental signal (50 Hz) of current. From this table it is noted that the amplitude was significantly improved after filtering at the 1 s time point by an estimated percentage of 66.47%. Also, at the 5 s time point the signal amplitude did not change before and after the filtering. However, at the time point of 9 s, it is observed that the amplitude of the signal after filtering is low compared to the amplitude of the signal before filtering, as this decrease is estimated at a rate of 8.96%.

**Table 9:** Ratios and values THD of current.

Times (s)	THD (%)		Ratios (%)
	Before harmonic current compensation	After harmonic current compensation	
1	56.87	0.32	99.43
5	43.05	0.29	99.32
9	27.50	0.26	99.05

**Table 10:** Signal amplitude values fundamental (50 Hz) of current.

Times (s)	Amplitudes (A)		Ratios (%)
	Before harmonic current compensation	After harmonic current compensation	
1	3373	10,060	66.47
5	10,060	10,060	0
9	11,040	10,050	8.96

In Table 11, the ripple values for *Ps* and *Qs* before and after filtering are given, where the ripples are given during three different loads. Also, the reduction ratio for *Ps* and *Qs* ripples is calculated. From this table, it is noted that the power ripples are lower after the filtering process compared to the ripples before the filtering, which proves that the proposed strategy is effective in reducing the value of *Ps* and *Qs* ripples, and this is what the calculated ratios show. In the first load, the reduction percentage was 13% and 52% for both *Ps* and *Qs*, respectively. In the third load, the ripple reduction ratio was 79% and 46% for both *Ps* and *Qs*, respectively. In addition, the lowest reduction percentages were in the first load and the largest reduction percentages were in the second load, where the percentages were estimated at 85% and 97% for both *Ps* and *Qs*, respectively.

**Table 11:** Ratios and values of ripples for DFIG power.

	*Ps* (W)			*Qs* (VAR)		
	Load 1	Load 2	Load 3	Load 1	Load 2	Load 3
Before filtering	92,066.5	522,899.8	323,719.87	190,674	394,019	143,362
After filtering	80,000	76,000	67,000	90,000	85,000	77,000
Ratios	13%	85%	79%	52%	97%	46%

### Techno-economic study of the proposed system

1. References^85–87^ focused on filtering the electrical grid when connecting non-linear loads. In^85,86^ utilized shunt active power filters, employing a dedicated system consisting of an inverter, a DC voltage source, and an *R-L* filter. On the other hand, in^87^ employed a different system, using an induction filter distribution transformer with a unique Dd0yn11 three-winding configuration.

It can be concluded that the approaches outlined in these papers entail significant costs to implement the studied systems.

The system we have developed is a self-system. Specifically, DFIG possess a unique characteristic wherein they can generate reverse harmonics that contribute to electric grid filtering. This is achieved using a single transformer, which also serves the purpose of power control. Therefore, it can be asserted that the implementation of this system, approaching a cost of 0$.

2. In^88–90^, the research relied on the utilization of DFIG to filter the electrical grid distorted by connecting non-linear loads. However, the loads used in their study had a relatively small current absorption from the electrical grid. It is known that DFIG cannot produce electric current above the nominal value. This limitation poses a challenge in dealing with loads of large absorption value.

Our work addresses this limitation by proposing a new algorithm. This algorithm enables the wind farm to effectively filter the electrical grid while simultaneously fulfilling its energy requirements. As a result, our approach is designed to work seamlessly with various forms of non-linear loads, accommodating their diverse energy characteristics, whether they absorb large or small currents.

To substantiate the credibility of the algorithm proposed in our work, which contributes to electrical grid filtering, we have created a comparative table (Table 12) with relevant literature:

**Table 12:** Comparative analysis of the performance of the proposed Self-filtering technique with literature.

References	Filtering method	Load current (A)	Before compensation THD (%)	After compensation THD (%)
^85^	SHAPF	50	35.84	4.38
^86^	p-q theory	20	29.18	3.19
	SRF theory		28.89	4.88
	Lyapunov function approach		30.40	1.61
	IRP Based approach		29.18	4.57
^87^	Traditional filtering method	–	–	14.12
	Inductive filtering method	–	–	4.09
^88^	PLL	10	17.34	5.68
	DFIG /APF			3.18
^89^	DFIG/APF	10	21.09	6.42
^90^	DFIG/APF	2	23.56	3.40
Proposed technique	Self-filtering—FS-MPC/WF	5000	56,87	0.32
		3062	43.05	0.29
		5270	27.50	0.26

In the case of non-linear loads that consume electricity more than the nominal power of the DFIG. In this case DFIG cannot participate in the filtering. Our proposal is to use wind farms to contribute to solving this problem by proposing a new algorithm.

Upon comparison, it is evident that our work is dedicated to filtering the electrical grid in the presence of non-linear loads with In/Substantial current absorption. The application of the proposed algorithm yielded favourable results, especially when dealing with the high current values of nonlinear loads (5000 A, 3026 A, and 5270 A).

On the other hand, the literature discussed in the table primarily addresses:

1. Non-linear loads with low current values (2–50 A).
2. The energy quality obtained in comparison to the loads used is also weak (1.61%-14.12).

The utilization of FS-MPC technique, known for enhancing both performance and power quality, can be summarized and compared with the literature in Table 13. Table 13 compares the proposed control with other research. The proposed method succeeded in testing robustness and reliability. FS-MPC technique increases performance compared to^52,67,69,91–96^, but its biggest benefit is the reduction of THD compared to^52,67,69,91–96^, which did not exceed 0.26–0.32%, indicating superior power quality.

**Table 13:** Benefits of employing FS-MPC technique in the proposed algorithm.

Publication paper	Tests		Performance				Power quality	Reliability	Robust
			Stability	Precision (%)	Rapidity RT (s)	Overshoot (%)	THD%		
^52^	Fuzzy_ SMC		Yes	–	0.32	–	–	–	–
^91^	PI		Yes	1.25	0.030	–	–	–	–
	RST		Yes	0.06	0.028	–	–	–	–
^92^	SMC-based Backstepping control		Yes	–	0.05	–	2.98	–	–
^93^	DTC		Yes	–	0.12	–	18.8	–	–
	FSC		Yes	–	0.16	–	8.26	–	–
	MPDC		Yes	–	0.15	–	8.17	–	–
^69^	FCS-MPC		Yes	–	0.15	–	6.12	–	–
^94^	FCS-MPC		Yes	–	0.12	–	4.29	–	–
^95^	FCS-MPC		Yes	0.11	0.11	–	0.49	–	–
^96^	FCS-MPC		Yes	–	–	–	7.22	–	–
^67^	FCS-MPC		Yes	–	0.045	–	$\approx 2$	–	–
Proposed technique	FS-MPC	PS	Yes	0.4	0.0247	0.02247	0.26–0.32	Yes	Yes
		QS		0.18	0.0234	0.03881			

## Conclusions

This paper introduces a management and control approach with self-filtering capabilities, taking into account fault ride-through considerations. The obtained results serve as compelling evidence affirming the efficacy of the proposed methodology. From these outcomes, the following conclusions can be drawn:

1. In the preliminary findings, the primary function of the wind farm is described, which involves injecting active and reactive energy into the grid while considering fault avoidance (LVRT) to protect the WF. Based on the achieved results, the management algorithm has demonstrated its efficacy in addressing three out of four operational modes. It successfully facilitates coordination among DFIG units, aligning with the requirements of the electrical grid. Moreover, the algorithm proves instrumental in safeguarding the WF against potential damage resulting from electrical grid faults by implementing effective isolation measures.
2. Addressing faults offers valuable insights into evaluating the effectiveness of diverse control strategies. In alignment with this principle, two types of faults, symmetric and asymmetric, were deliberately introduced at the PCC level. The outcomes of these fault simulations clearly demonstrated the efficiency of the proposed control system in effectively controlling both symmetric and asymmetric faults. These results underscore the capability of the proposed control strategy to enhance the overall performance of the studied system in the face of fault conditions.
3. To achieve the hybrid functionality of WESC/self-filtering, it is essential to determine the current consumption of the nonlinear load, identify the farm units contributing to the process, and subsequently set the references for harmonic currents in the electrical grid. In this section, the proposed method yields promising results.

After integrating linear loads, the THD value of grid currents ranges from 27.52 to 56.87%. However, the utilization of the proposed supervision unit with FS-MPC technique, working in harmony, results in improved energy quality (the THD values are found in the reduced range from 0.27 to 0.32%), establishing a robust system characterized by high reliability and superior performance.

Nonetheless, it is worth noting that various scenarios must be considered, such as energy compensation in the event of a units from a WF becoming isolated due to a malfunction. This aspect will be addressed in future discussions. Building upon the insights from^97^, emphasis can be placed on the contribution of wind turbines in Hybrid DC Micro Grids to enhance system stability and improve voltage and energy management dynamics within a hybrid system. Our work represents a transitional stage, aiming to introduce WFs into hybrid systems and coordinate them effectively to contribute to system stabilization.

As future work, this work will be carried out experimentally and the results obtained will be compared with existing works in terms of current and power quality. New strategies based on combining non-linear controls will also be applied to control a group of turbines in a wind farm.

## Data Availability

Data available on request from the authors. The datasets used and/or analysed during the current study available from the first and third authors on reasonable request. In the event of communication, the first and third authors (Abdelkader Achar and Habib Benbouhenni, E-mail: a.achar1@yahoo.com, habib.benbouenni@nisantasi.edu.tr) will respond to any inquiry or request.

## Abbreviations

PWM: Pulse width modulation
DPC: Direct power command
VC: Vector command
PC: Predictive control
SVM: Space vector modulation
PF: Power factor
WP: Wind power
WS: Wind speed
DTC: Direct torque command
SSE: Steady-state error
MPC: Model predictive control
WF: Wind farm
DFIG: Doubly-fed induction generator
GSC: Gird side converter
RSC: Rotor side converter
NN: Neural network
SMC: Sliding mode command
SC: Synergetic command
FOC: Field-oriented command
BC: Backstepping command
PLL: Phase-locked loop
HC: Hysteresis comparator
STA: Super-twisting algorithm
AI: Artificiel intelligent
MFPCC: Model-free predictive current control
IOFL: Input–output feedback linear
FCS-MPC: Finite-control-set model predictive control
PFR: Primary frequency response
PI: Proportional-integral controller
*Ps*: Active power
*Is*: Stator current
*Ir*: Rotor current
*Qs*: Reactive power
